# Finite dimensional state representation of physiologically structured populations

**DOI:** 10.1007/s00285-019-01454-0

**Published:** 2019-12-21

**Authors:** Odo Diekmann, Mats Gyllenberg, Johan A. J. Metz

**Affiliations:** 1 Department of Mathematics, University of Utrecht, P.O. Box 80010, 3508 TA Utrecht, The Netherlands; 2 Department of Mathematics and Statistics, University of Helsinki, P.O. Box 68, 00014 Helsinki, Finland; 3 Mathematical Institute and Institute of Biology, Leiden University, 2333 CA Leiden, The Netherlands; 4 Evolution and Ecology Program, International Institute of Applied Systems Analysis, 2361 Laxenburg, Austria

**Keywords:** ODE-reducibility, Linear chain trick, Evolutionary system, Input–output system, 92D25, 93B11

## Abstract

In a physiologically structured population model (PSPM) individuals are characterised by continuous variables, like age and size, collectively called their i-state. The world in which these individuals live is characterised by another set of variables, collectively called the environmental condition. The model consists of submodels for (i) the dynamics of the i-state, e.g. growth and maturation, (ii) survival, (iii) reproduction, with the relevant rates described as a function of (i-state, environmental condition), (iv) functions of (i-state, environmental condition), like biomass or feeding rate, that integrated over the i-state distribution together produce the output of the population model. When the environmental condition is treated as a given function of time (input), the population model becomes linear in the state. Density dependence and interaction with other populations is captured by feedback via a shared environment, i.e., by letting the environmental condition be influenced by the populations’ outputs. This yields a systematic methodology for formulating community models by coupling nonlinear input–output relations defined by state-linear population models. For some combinations of submodels an (infinite dimensional) PSPM can without loss of relevant information be replaced by a finite dimensional ODE. We then call the model ODE-reducible. The present paper provides (a) a test for checking whether a PSPM is ODE reducible, and (b) a catalogue of *all* possible ODE-reducible models given certain restrictions, to wit: (i) the i-state dynamics is deterministic, (ii) the i-state space is one-dimensional, (iii) the birth rate can be written as a finite sum of environment-dependent distributions over the birth states weighted by environment independent ‘population outputs’. So under these restrictions our conditions for ODE-reducibility are not only sufficient but in fact necessary. Restriction (iii) has the desirable effect that it guarantees that the population trajectories are after a while fully determined by the solution of the ODE so that the latter gives a complete picture of the dynamics of the population and not just of its outputs.

## Introduction

From the very beginning of community modelling, ordinary differential equations (ODEs) have been its main tool. This notwithstanding the fact that much earlier Euler (1760) and other mathematicians working on population dynamics had already considered age structured models, see (Bacaër 2008, 2011; Gyllenberg 2007) for more information on the history of population dynamics. This probably had two causes, the architects of the initial flurry in the nineteen-twenties and thirties (cf. Scudo and Ziegler 1998) and their successors like MacArthur and May (cf. Kingsland 1995) got their inspiration from the successes of physics, which is dominated by differential equations, and ODEs are rather easier to write down and analyse than e.g. integral equations. However, the assumptions needed to arrive at ODEs generally match biological reality less closely, and give these models more of a toy character: good to get new ideas, but difficult to match in some detail to concrete ecological systems. That for the latter age may well matter also mathematicians know from their immediate experience: few women give birth before the age of ten and while most humans nowadays reach their seventy’s anniversary still few live beyond a century. For this reason, many mathematical modellers turned to age as a structuring variable, even in the non-linear realm. However, for ectotherms, that is, all organisms other than mammals and birds, size usually matters far more than age (cf. de Roos and Persson 2013). We have spent considerable effort in the past to develop tools for studying general physiologically structured models in the hope to gradually overcome the surviving endotherm-bias of the modelling community. Yet ODE models remain paramount as didactical tools and for the initial exploration of so far unexplored mechanisms, notwithstanding the disadvantage that in these models individual level mechanisms generally can only be fudged instead of faithfully represented. Given this situation, it becomes of importance to explore in what manner ODE models fit among the physiologically structured ones. Of course, there is the boringly simple embedding of the unrealistic case where individuals indeed have only a single, or at most a few possible states.

### Example 1.1

Consider a size-structured population with individual size (biomass) denoted as *x*, starting from a birth size $x_b$, individual growth rate *g*(*x*, *E*), *E* a resource density, per capita birth rate $\beta (x,E)$, and per capita death rate $\mu (x,E)$. For such populations, if

1.1 $$\begin{aligned} \frac{x_b \beta (x,E) + g(x,E)}{x} - \mu (x,E)=v(E), \end{aligned}$$

the population biomass *B* per unit of spatial area or volume (below to be abbreviated as just volume) satisfies

1.2 $$\begin{aligned} \frac{dB}{dt} = v(E)B, \end{aligned}$$

To see this, observe that the left hand side of (1.1) corresponds to the contribution to the change in population biomass by an individual of size *x* expressed as fraction of its own biomass. So if we integrate this term over the biomass density over size (and volume), say *xn*(*t*, *x*), *n* the numeric (per unit of volume) size density, we get the total change in biomass (per unit of volume), (cf. de Roos et al. 2013).

If moreover

(i) the per capita contribution to the “consumptive pressure on a resource unit” is a product of an individual’s size and a size-independent functional response based component, say, *f*(*E*) / *E*,
(ii) all other populations in the community are similarly affected only by our focal population’s biomass, and
(iii) we ourselves are also only interested in this quantity,

then we can for all practical purposes represent our focal population by no more than its biomass.

We call (1.2) an ODE-reduction of the size-structured population model.

The question then naturally arises whether or not this example of ODE-reducibility is essentially the only one, that is, up to coordinate choices, such as in the case of isomorphs not biomass but its scaled cubic root, length. The following example shows this not to be the case.

### Example 1.2

*Daphnia models.* Now let in the wake of (Kooijman and Metz 1984) and (de Roos et al. 1990) size be represented by length, starting from a size $x_b$ at birth, the growth rate be given by $g(x,E)=\delta f(E) -\varepsilon x$, the per capita birth rate by $\beta (x,E)=\alpha f(E)x^2$, the per capita death rate by $\mu$, and the per capita resource consumption by $f(E)x^2$. (This means that individual biomass, *w*, scaled to be equal to $x^3$, grows as $3\delta f(E)w^{2/3}-3\varepsilon w$, that is, mass intake is taken to be proportional to surface area and metabolism to biomass.) Let *n*(*t*, *x*) again denote the numeric size-density. Now define

1.3 $$\begin{aligned} N_i (t) = \int \limits _{x_b}^{x_{\max } } {x^i } n(t,x)dx, \end{aligned}$$

that is, $N_0$ is the total population size, $N_1$ the total population length, $N_2$ the total population surface area, $N_3$ the total population biomass, all per unit of volume. Then

1.4 $$\begin{aligned} \frac{dN_0}{dt}= & {} \alpha f(E)N_2 - \mu N_0, \nonumber \\ \frac{dN_1}{dt}= & {} x_{b} \alpha f(E)N_2 + \delta f(E)N_0 - (\mu + \varepsilon )N_1, \nonumber \\ \frac{dN_2}{dt}= & {} x^2_{b} \alpha f(E)N_2 + 2\delta f(E)N_1 - (\mu + 2\varepsilon )N_2, \nonumber \\ \frac{dN_3}{dt}= & {} x^3_{b} \alpha f(E)N_2 + 3\delta f(E)N_2 - (\mu + 3\varepsilon )N_3, \end{aligned}$$

etc.. If the only other component of the community is an unstructured resource, and we need no further output from the population than its total biomass per unit of volume, we can combine (1.4) with

1.5 $$\begin{aligned} \frac{dE}{dt} = h(E) - f(E)N_2 \end{aligned}$$

into a sufficient representation of our community model.

The differential equation for $N_0$ is obvious, and so is the first term in the differential equation for $N_1$. To understand the second term observe that *g* consists of two terms, the first of which is size independent. This term makes all individuals of the population increase their length at a rate $\delta f(E)$. We get the corresponding increase in the total population length by multiplying this $\delta f(E)$ with the total population density. The $\varepsilon$ in the last term also derives from the length growth except that the corresponding term in *g* contains a factor *x*. When we account for this *x* when calculating the integral over *n* we get $N_1$. The differential equations for the other $N_i$ follow in a similar manner.

ODE-reducibility of age-structured models and, slightly more generally, of distributed delay systems, has been investigated since the mid 1960s (Vogel 1965; Fargue 1973, 1974; Gurtin and MacCamy 1974, 1979a, b; McDonald 1978, 1989).

It has already been known for a long while that there also exist more realistic cases, where for instance a size-structured model allows a faithful representation in ODE terms (Murphy 1983; Cushing 1989; Metz and Diekmann 1991).

The next question is then whether we can characterise the set of *all* possible cases. For the practically important subset of cases where the population birth rate figures on the list of population outputs and with a single state variable on the level of the individuals, the last author solved this problem on a heuristic level already in 1989 during a holiday week in summer spent at the Department of Applied Physics of the University of Strathclyde. An allusion to this was given in a “note added in print” to the paper (Metz and Diekmann 1991). However, it took till now before we together had plugged all the minor holes in the proof. Below you find the result.

## Preview of Sects. 3 to 6

In this section we give a preview of the main content of the paper, first for theoretical biologists and probabilists and then for all kinds of mathematicians. The much shorter paper (Diekmann et al. 2019) provides additional examples and may serve as a more friendly user guide to ODE-reducibility of structured population models.

### Mainly for theoretical biologists and probabilists: the context of discovery

#### Biological context

The term “physiologically structured population models” (PSPM) refers to large system size limits of individual-based models where (i) individuals are differentiated by physiological states, e.g. $x = (\mathrm {size, age})$, referred to as i(ndividual)-states, (ii) the world in which these individuals live is characterized by a set of variables collectively called environmental condition, to be denoted as *E*. [Hard proofs for the limit conjectures are still lacking except for age-based models (cf. Tran 2008, and the references therein), and more recently also for a class of simple (size,age)-based ones (Metz and Tran 2013).] Sections 2.2 and 3 go into how these deterministic models can be specified by means of equations.

The i-level model ingredients are a set of feasible i-states, $\varOmega \subset \mathbb {R}^m$, $m \in {\mathbb {N}}$, and, most commonly,

(i) a rate of i-state change taken to be deterministic, *g*(*x*, *E*),
(ii) a death rate, $\mu (x,E)$, and
(iii) a birth rate, $\beta (x,E)$.

In the general case $\beta (x,E)$ has as value a distribution over $\varOmega$. However, from a mathematical perspective it is preferable to use instead of “distribution” the term “measure” as this is more encompassing, and the birth rate does not total to one and may consist of a mixture of discrete and continuous distributions. (Actually, we should even be a bit more general and talk about a signed measure as a cell that divides generates a measure over the states where the daughters may land plus a compensating negative mass, equal to minus the division rate, at the state of the mother.)

*Notational convention* The value of $\beta (x,E)$ for the measurable set $\omega \subset \varOmega$ is denoted by $\beta (x,E,\omega )$. A similar convention applies to other measure valued functions.

The p(opulation)-state then is a measure *m* on $\varOmega$. However, on many occasions it suffices to think in terms of just densities *n* on $\varOmega$, or $n\in L^1(\varOmega )$ in the mathematicians’ jargon. As a consequence of how the rates are specified, when *E* is given as a function of time (below to be looked at as input) the individuals are independent (except for a possible dependence of their birth state on the state of their parents), and hence the dynamics of the p-state is linear.

The more interesting case is when *E* is determined by the surrounding community. Community models are sets of population models coupled through a common *E*. This leads to c(ommunity)-state spaces that are products of the state spaces of the comprised species, times the state spaces of any non-living resources. The mass action principle tells that generally *E* can be calculated by applying a linear map to the c-state, like when a predation pressure equals the sum of the predation pressures exerted by all individuals in the community. This leads us to the final set of ingredients of a population model:

(iv) functions of (*x*, *E*), like biomass or per capita feeding rate, that when cumulated over all individuals produce components of the population output.

Side remarks on terminology: In our context, each output component is thus obtained by taking the integral of the p-state over $\varOmega$ after multiplying it with a, possibly *E*-dependent, function of the i-state variables. This function specifies the relationship between the i-state and the property that we want to measure, e.g., biomass as a function of length. In mathematical jargon we say that the output components are obtained by applying a linear functional, i.e., a linear map from the p-state space to the real numbers. The corresponding function will be referred to as weight function, and for a p-state *m* and weight function $\psi$, the corresponding map will be written as $\left\langle {m,\psi } \right\rangle$.

The population dynamical behaviour of individuals almost never can be captured in terms of a finite number of i-states. Yet, ecological discourse is dominated by ODE models. This leads to the philosophical question which of these models can be justified from the more realistic physiologically structured populations perspective. At the more pragmatic side there is moreover the problem that in community biology PSPM usually become too difficult to handle for more than two or three species. This leads to the complementary question whether there are relevant choices of model ingredients for which a PSPM can be represented by a low dimensional system of ODEs. To answer these questions we looked at populations as state-linear input–output relations, with *E* as input, and as output a population’s contribution to *E* as well as anything that ‘a client’ may want to keep track of. The key question addressed in this paper is thus: under what conditions on the model ingredients is it possible to obtain the same input–output relation when the PSPM is replaced by a finite dimensional ODE? (This representation may have an interpretation in its own right, but this is not required.) If such a representation is possible, we say that the population model is reduced to the ODE or that the input–output relation is realised by it.

#### The mathematical question

Our starting point thus are models that can be represented as in the following diagram.

**Fig. 1:**
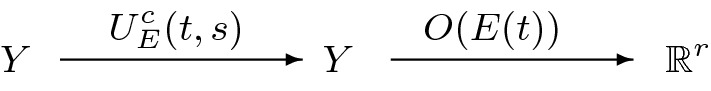
Structure of models with output

In Fig. 1*Y* is the p-state space and ${\mathbb {R}}^r$ the output space. *E* is the time course of the environment and $U_E^c (t,s)$ the (positive) linear state transition map with $s,\; t$ the initial and final time. (The upper index *c* here refers to the mathematical construction of the p-state, explained in Sect. 3, through the cumulation of subsequent generations.) Finally *O*(*E*(*t*)) is the linear output map. The mathematical question then is under which conditions on the model ingredients it is possible to extend the diagram in Fig. 1 (for all $E,\; t,\; s$) to the following diagram.

Here *P* is a linear map, $\varPhi _E (t,s)$ a linear state transition map (which should be differentiable with respect to *t*) and *Q*(*E*(*t*)) a linear output map. The dynamics of the output cannot be generated by an ODE when the space spanned by the output vectors at a given time is not finite dimensional. Hence ODE reducibility implies that there exists an *r* such that the outputs at a given time can be represented by ${\mathbb {R}}^r$. (Below we drop the time arguments to diminish clutter, except in statements that make sense only for each value of the argument separately, or when we need to refer to those arguments.) Moreover, the biological interpretation dictates that

$$\begin{aligned} O(E)m= \left\langle {m,\varGamma (E) } \right\rangle :=\int _\varOmega \varGamma (E)(x)m(dx), \end{aligned}$$

where *m* is the p-state and the components of the vector $\varGamma (E)$ are functions $\gamma _i(E): \varOmega \rightarrow \mathbb {R}$.

Thanks to the linearity of $U^c_E (t,s)$ and *O*(*E*(*t*)) we can without loss of generality assume *P*, $\varPhi _E (t,s)$ and *Q*(*E*(*t*)) to be linear. Moreover, ODE reducibility requires that *P* can be written as $Pm = \left\langle {m,\varPsi } \right\rangle$ with $\varPsi =(\psi _1,\dots ,\psi _k)^\mathrm {T}$, $\psi _i:\varOmega \rightarrow \mathbb {R}$, where the $\psi _i$ should be sufficiently smooth to allow

2.1 $$\begin{aligned} {{dN} \big / {dt}} = K(E)N,\; \mathrm {with} \; N:=Pm\; \mathrm {and} \; K(E(t)):= \left. {{{d\varPhi _{E} (t,s)} \big /{dt}}} \right| _{s= t}. \end{aligned}$$

(The last expression comes from combining ${{d\varPhi _E (t,s)} \big /{dt}} = K(E(t))\varPhi _E (t,s)$ and $\varPhi _E (t,t)=I$.) Finally, we should have $O(E)=Q(E)P$, and therefore $\varGamma (E)=Q(E)\varPsi$, implying that the output weight functions should be similarly smooth. (The precise degree of smoothness needed depends on the other model ingredients in a manner that is revealed by the TEST described in Sect. 2.1.4.)

#### A tool

The main tool in the following considerations is the so-called backward operator, to be called $\bar{A}(E)$, as encountered in the backward equation of Markov process theory and thereby in population genetics, used there to extract various kinds of information from the process without solving for its transition probabilities. What matters here is that the backward operator summarises the behaviour of the “clan averages” $\bar{\psi } (t,s)$ of weight functions $\psi$ (such as occur in $Pm = \left\langle {m,\varPsi } \right\rangle$), defined by

2.2 $$\begin{aligned} \bar{\psi } (t,s)(x ) := \int _\varOmega \psi (\xi )m(t,s;\delta _x,d\xi ), \end{aligned}$$

where ${m(t,s;\delta _{x } )}$ is the p-state resulting at *t* from a p-state corresponding to a unit mass $\delta _x$ at *x* at time *s* (hence the term clan average), in the sense that

2.3 $$\begin{aligned} \bar{A}(E(s)) \bar{\psi }(t,s) = -\frac{{\partial \bar{\psi }(t,s)}}{{\partial s}}. \end{aligned}$$

For further use we moreover note that

2.4 $$\begin{aligned} \bar{\psi } (s,s) =\psi , \end{aligned}$$

independent of *s*, which on differentiating for *s* gives

2.5 $$\begin{aligned} \mathrm {at}\; t=s : \quad -\frac{{\partial \bar{\psi } }(t,s)}{{\partial s}} = \frac{{\partial \bar{\psi } }(t,s)}{{\partial t}}. \end{aligned}$$

(When *E* does not depend on *s*, (2.5) also holds good for $t\ne s$, leading to the perhaps more familiar form of the backward equation: $d\bar{\psi } /dt = \bar{A}\bar{\psi }$.)

One reason to start from (2.2) is that it leads to simple interpretation-based heuristics for calculating backward operators, which we shall discuss a little later on. (More abstract and rigorous functional analytical counterparts to the intuitive interpretation-based line of argument developed here can be found in Sects. 2.2 and 4.) The reason for coming forth with the backward operator is that it provides the counterpart in the space of weight functions $\psi$ of the time differentiation in (2.1). To see that, the perspective sketched so far still needs a slight extension. A look at (2.2) shows that we can also interpret the ${\bar{\psi }}(t,s)$ as weight functions in their own right that can be paired with $\delta _x$ as $\left\langle {\delta _x,\bar{\psi } (t,s) } \right\rangle \,(\,=\bar{\psi } (t,s)(x )\,)$. Moreover, thanks to the linearity of (2.2), (2.3) and the p-state transition maps $U_E^c (t,s)$ and the consequent linearity of $m(t,s;\cdot )$, we can extend the action of these candidate functionals to more general measures $m_s$ at time *s*, and in this way calculate $\int _\varOmega {\psi (\xi )m(t,s;m_s,dx)}$ as $\left\langle {m_s,\bar{\psi } (t,s) } \right\rangle$. This sleight of hand transforms the question about the change of *Pm* over time to a question about the dynamics of the $\bar{\psi }(t,s)$, for which we can find the time derivative by applying $\bar{A}(E)$ to $\bar{\psi }$. The final step then is to make the connection with (2.1) by setting $s=t$ and using (2.4), to arrive at

2.6 $$\begin{aligned} \bar{A}(E)\varPsi =K(E)\varPsi . \end{aligned}$$

To apply these ideas we need to find expressions for the backward operators. To this end we use that we assumed *E* to be given, making individuals independent. Then on the level of population averages it makes no difference whether we start with a scaled large number of individuals all with i-state *x* or with a single individual in that state. Since it is easier to think in terms of individuals, we shall do the latter. We can then look at the effect on the clan averages of the founder individual engaging in each of the component processes (i) to (iii) over a short time interval from *s* to $s+h$. For such short intervals the effects of the interaction of the components in determining their combined effect on the clan average is only second order in *h* and can be neglected together with the other higher order terms. Hence the backward operator can be written as a sum of operators corresponding to the model ingredients as introduced at the beginning of Sect. 2.1.1. As this is useful for the remainder of the paper we here combine ingredients (i) and (ii):

2.7 $$\begin{aligned}&\bar{A}(E)=\bar{A}_0 (E)+\bar{B}(E), \nonumber \\ {\mathrm {(i)\; and\; (ii)\!:}} \quad&\left( {\bar{A}_0 (E){\psi }} \right) (x ) = \frac{{{\text {d}}{\psi } }}{{{\text {d}}x }}(x)g(x,E) - \mu (x,E){\psi } (x ),\nonumber \\ {\mathrm {(iii)\!:}} \quad&\left( {\bar{B}(E){\psi } } \right) (x ) = \int \limits _\varOmega {{\psi } (\xi )\beta (x,E,d\xi )}. \end{aligned}$$

The expressions in (2.7) are found as follows:

(i) If we neglect births and deaths, for small *h* an individual situated at *x* at time *s* produces an individual situated at $x+g(x, E(s))h$ at time $s+h$. Therefore ${\bar{\psi }}(t,s+h)(x+g(x, E(s))h)={\bar{\psi }}(t,s)(x)$, which after on both sides subtracting $\bar{\psi }(t,s+h)(x)$ and dividing by *h*, gives
  2.8 $$\begin{aligned} \left. {-\frac{{\partial \bar{\psi }}(t,s)(x )}{{\partial s}}} \right| _{{\text {movement}}} = \frac{{\partial \bar{\psi } }(t,s)(x )}{{\partial x }}g(x,E(s)). \end{aligned}$$
(ii) Next we account for the possibility that individuals die on the way, which happens with probability $\mu (x,E(s))h$, so that, if we neglect movement and births, ${\bar{\psi }}(t,s)(x)=(1-\mu (x,E(s))ds){\bar{\psi }}(t,s+h)(x)$ (over a longer time survival is less), giving
  2.9 $$\begin{aligned} \left. {-\frac{{\partial \bar{\psi } }(t,s)(x )}{{\partial s}}} \right| _{{\text {death}}} = -\mu (x,E(s)){\bar{\psi }}(t,s)(x ). \end{aligned}$$
(iii) Finally a parent at *x* through its kids at $\xi$ will have added a contribution $h\beta (x,E(s),d\xi ){\bar{\psi }}(t,s)(\xi )$ to ${\bar{\psi }}(t,s)(x)$, which is missing in $\bar{\psi }(t,s+h)(x)$. Summing over all these contributions gives
  2.10 $$\begin{aligned} \left. {-\frac{{\partial \bar{\psi } }(t,s)(x )}{{\partial s}}} \right| _{{\text {births}}}=\int _\varOmega {\bar{\psi }}(t,s)(\xi )\beta (x,E(s),d\xi ). \end{aligned}$$

#### Testing combinations of model ingredients for ODE reducibility

The classical search heuristic for finding a state space representation for a given set of dynamical variables in continuous time is to see whether their derivatives can be expressed in terms of the variables themselves, and, if not, to join the derivatives for which this is not the case as additional prospective state variables to the original set, whereupon the procedure is repeated till one succeeds or runs out of patience (cf. Diekmann et al. 2018, p. 1443; Fargue 1973).

If one is after a representation as a state linear system with a linear output map, “can be expressed as” translates to “is linearly dependent on”, a property that can be checked algorithmically, so that with infinite patience we end up with a firm conclusion. In our case the only difference is that we choose not to look at the output variables themselves, but at the weight functions by which they are produced from the population state, and therefore replace the time derivative of the output variables by the backward operator applied to these functions.

*Notation* For the remainder of this subsection we shall again use *E* to denote the environmental conditions, as opposed to the course of the environment as we did in the previous two subsections.

- TEST
- Choose a basis $V_0=\{\psi _1,\dots ,\psi _{k_0}\}$ for the $\gamma _i$ making up the population output map.
- Provided that the expression $\bar{A}(E)V_i$ makes sense, extend $V_{i}$ to a basis $V_{i+1}$ for $V_i \cup \bigcup _{\mathrm{{all \; possible \; }}E} {\bar{A}(E)V_{i} }$.
- The population model is ODE reducible if and only if the expressions $\bar{A}(E)V_i$ keep making sense, and the $V_i$ stop increasing after a certain $i=h$.

For the $\bar{A}(E)$ from (2.7), $\bar{A}(E)V_i$ makes sense iff all elements of $V_i$ are differentiable. (For a mathematically more precise rendering see Sect. 4.)

If the biological ingredients of a model satisfy the above test, it is possible to go directly from the ingredients to the ODE.

##### Example 2.1

*Daphnia models, continued.* In Example 1.2 the output weight functions were $x^3$ and $x^2$. Applying the backward operator

$$\begin{aligned} \bar{A}(E)\psi (x)= (\delta f(E) -\varepsilon x)\frac{d{\psi }}{dx}(x) - \mu {\psi } (x )+ {{\psi } (x_b)\alpha f(E)x^2 } \end{aligned}$$

to these functions gives

2.11 $$\begin{aligned} \bar{A}(E)\left( {\begin{array}{*{20}c}{x^3 } \\ {x^2 } \\ \end{array}} \right)= & {} \left( {\begin{array}{*{20}c} {(\delta f(E) - \varepsilon x)3x^2 - \mu x^3 + x_b^3 \alpha f(E)x^2 } \\ {(\delta f(E) - \varepsilon x)2x \;\; - \mu x^2 + x_b^2 \alpha f(E)x^2 } \\ \end{array}} \right) \nonumber \\= & {} \left( {\begin{array}{*{20}c} { x^3_b \alpha f(E)x^2 + 3\delta f(E)x^2 - (\mu + 3\varepsilon )x^3 } \\ {x^2_b \alpha f(E)x^2 + 2\delta f(E)x \;\;- (\mu + 2\varepsilon )x^2} \\ \end{array}} \right) . \end{aligned}$$

This introduces *x* as an additional weight function, linearly independent of $x^3$ and $x^2$. Applying the backward operator to *x* gives

2.12 $$\begin{aligned} \bar{A}(E)x=\delta f(E) - \varepsilon x - \mu x +x_b \alpha f(E)x^2= x_b \alpha f(E)x^2 + \delta f(E) - (\mu + \varepsilon )x,\nonumber \\ \end{aligned}$$

introducing the constant 1 as further weight function. Applying $\bar{A}(E)$ to 1 gives

2.13 $$\begin{aligned} \bar{A}(E)1= - \mu +x_b \alpha f(E)x^2= x_b\alpha f(E)x^2 - \mu , \end{aligned}$$

which introduces no further linearly independent weight functions, and thus ends the process. Hence, a *P* can be built from the weight functions 1, *x*, $x^2$ and $x^3$ leading to the four dimensional ODE representation already derived in Example 1.2.

Integrating (2.11) to (2.13) left and right over the p-state gives (1.4). This is why we could reorder (2.11) to (2.13) to look like (1.4), with the $N_i$ replaced by $x^i$. Rewritten in matrix form this then gives the *K*(*E*) from (2.1). Note though that in the process we have made some particular choices in order to get the precise form (1.4). In general *K*(*E*) is only unique up to a similarity transform, corresponding to alternative choices of a basis for the weight functions $\psi _1,\dots ,\psi _k$, with a corresponding change of *Q*(*E*).

##### Remark

At first sight the TEST may not seem very practical as deciding that a certain set of model ingredients is not ODE reducible may require infinitely many operations. However, if the model ingredients come in the form of explicit expressions it can often be inferred from these whether the combination of ingredients will or will not pass the test. And even when those expressions on first sight are less than transparent, not all is lost, as in practice people are generally not so much interested in whether a certain model is ODE reducible as such, but in whether there exists a representation with a relatively low dimensional state space, which after specification of the maximum allowed dimension leads to a task executable by e.g. Maple^TM^ or Mathematica^®^.

#### A catalogue of ODE reducible models

Modellers often initially still have quite some freedom in their choice of model ingredients. Hence it is useful to construct catalogues of classes of ODE reducible models, to help them make their choices with an eye on future model tractability (for a good example, see (Cushing 1989)). For this purpose observe that the TEST is no more than an attempt to construct a vector $\varPsi$ of weight functions such that for all environmental conditions *E* (i) $\varGamma (E)=Q(E)\varPsi$ and (ii)

2.14 $$\begin{aligned} \bar{A}(E)\varPsi = K(E)\varPsi , \end{aligned}$$

suggesting the following strategy: start from some specific promising model class, and within this class try to solve (2.14) for $\varPsi$ and *K*. In Sects. 5 and 6 we concentrate on one particular class of models that is both hallowed by tradition, and has the good property of allowing the p-state trajectory to be reconstructed by relatively easy means:

2.15 $$\begin{aligned} \beta (x,E) = \sum \limits _{i = 1}^p{\beta _i (E)} \alpha _i (x) \end{aligned}$$

with the $\alpha _i$ treated as output weight functions au par with the other $\gamma _i$. (For the numerics it helps when the $\beta _i(E)$ consist of just a few point masses at positions that depend smoothly on *E*.) For the remainder of this subsection we assume that (2.15) holds good.

##### Remark

In earlier papers like (Metz and Diekmann 1991) we referred to ODE reducible models satisfying (2.15) as ordinarily ODE reducible or ODE reducible *sensu stricto*, as these models then were the only ones figuring in discussions of ODE reducibility (or linear chain trickability as it then was called).

The assumption that the $\alpha _i$ are additional output weight functions makes that we can absorb the birth term of the backward operator into the right hand side of (2.14) giving

2.16 $$\begin{aligned} \bar{A}_0(E)\varPsi =H(E)\varPsi . \end{aligned}$$

All our general results so far pertain to the case where $\mathrm {dim}(\varOmega )=1$. In Sect. 8 we show how the results for this case can be combined to make ODE reducible models with $\mathrm {dim}(\varOmega )>1$, but a full catalogue for general $\varOmega$ is still lacking. So for the remainder of this subsection we assume that $\mathrm {dim}(\varOmega )=1$.

Below we give a gross sketch of the reasoning. As first step we choose a fixed constant $E=E_\mathrm {r}$, write $v_\mathrm {r}(x)=g(x,E_\mathrm {r}),\, \mu _\mathrm {r}(x)=\mu (x,E_\mathrm {r}),\, H_\mathrm {r}=H(E_\mathrm {r})$, where the label $\mathrm {r}$ in the subscipts stands for “reference”, and solve the corresponding version of (2.16),

2.17 $$\begin{aligned} \frac{d\varPsi }{dx}(x)v_\mathrm {r}(x)-\mu _\mathrm {r}(x)\varPsi (x)=H_\mathrm {r}\varPsi (x), \end{aligned}$$

for $\varPsi$. The result is that $\varPsi (x)$ is the product of

$$\begin{aligned} {\mathscr {G}}_\mathrm {r}(x):=\exp \left( \int ^x \frac{\mu _\mathrm {r}(\xi )}{v_\mathrm {r}(\xi )}d\xi \right) \end{aligned}$$

(note that ${\mathscr {G}}_\mathrm {r}(x)$ can be interpreted as the inverse of a survival probability up to size *x*) and a matrix exponential in the transformed i-state variable

$$\begin{aligned} \zeta _\mathrm {r}(x):=\int ^x\frac{d\xi }{v_\mathrm {r}(\xi )}. \end{aligned}$$

This tells that the weight functions $\psi$ should be linear combinations of polynomials times exponentials in $\zeta _\mathrm {r}$, all multiplied with the same ${\mathscr {G}}_\mathrm {r}$. The fact that these weight functions should not depend on *E* then gives a set of conditions that should be satisfied by the $\varPsi$, *g* and $\mu$ together. There are three possibilities to satisfy these conditions:

(i) and (ii) Without any restrictions on $H_\mathrm {r}$, the death rate $\mu$ should be decomposable as $g(x,E)\gamma _0(x)+\mu _0(E)$, that is, a death rate component that at each value of *x* depends on how fast an individual grows through this value plus a death rate component that does not depend on *x*, and

(i) the representation should be one-dimensional, in which case $\varPsi (x) = d{\mathscr {G}}(x)$ with ${\mathscr {G}}(x):=\exp \left( \int ^x \gamma _0(\xi )d\xi \right)$, *d* a scalar of choice, and $H(E)=\mu _0(E)$,
  or, in the higher dimensional case,
(ii) *g* should be decomposable as $g(x,E) = v_0(x)v_1(E)$, so that we may interpret $\zeta (x):=\int ^x(v_0(\xi ))^{-1}d\xi$ as physiological age. *H* in this case can be written as $H(E) = -\mu _0(E)I + v_1(E) L$, with *L* an arbitrary matrix with all eigenvalues having geometric multiplicity one. $\varPsi$ can then be written as
  $$\begin{aligned} \varPsi (x)={\mathscr {G}}(x)D\left( e^{\lambda _1 z},\dots ,z^{k_1-1} e^{\lambda _1 z},\dots , e^{\lambda _r z},\dots ,z^{k_r-1} e^{\lambda _r z} \right) ^\mathrm {T}, \end{aligned}$$
  *D* a nonsingular matrix, with a corresponding representation of *L* as
  $$\begin{aligned}L= D\left( {\begin{array}{*{20}c} {\left( {\begin{array}{*{20}c} {\lambda _1 } &{}\quad {} &{}\quad {} &{}\quad 0 \\ 1 &{}\quad \ddots &{}\quad {} &{}\quad {} \\ {} &{}\quad \ddots &{}\quad \ddots &{}\quad {} \\ 0 &{}\quad {} &{}\quad {k_1 - 1} &{}\quad {\lambda _1 } \\ \end{array} } \right) } &{}\quad {} &{}\quad 0 \\ {} &{}\quad \ddots &{}\quad {} \\ 0 &{}\quad {} &{}\quad {\left( {\begin{array}{*{20}c} {\lambda _r } &{}\quad {} &{}\quad {} &{}\quad 0 \\ 1 &{}\quad \ddots &{}\quad {} &{}\quad {} \\ {} &{}\quad \ddots &{}\quad \ddots &{}\quad {} \\ 0 &{}\quad {} &{}\quad {k_r - 1} &{}\quad {\lambda _r } \\ \end{array} } \right) } \\ \end{array} } \right) D^{ - 1}. \end{aligned}$$
(iii) For higher dimensional representations also slightly less restricted growth laws are possible, but at the cost of a severe restriction on the eigenvalues of $H_\mathrm {r}$ which should lie in special regular configurations in the complex plane. A lot of hard work is then required to render the corresponding class of representations into a biologically interpretable form. The end result becomes a slight extension of the Daphnia model of Examples 1.2 and 2.1 with $\mu (x,E) = g(x,E)\gamma _0(x) + \mu _0(E)$, $g(x,E) = v_0(x)(v_1(E)+v_2(E)\zeta )$ (note that if we transform from *x* to $\zeta$ the growth law stays the same but for the disappearance of the factor $v_0(x)$). In this case $\varPsi (x) = {\mathscr {G}}(x)D\varXi (x)$ with $\varXi (x) = (1,\ldots , \zeta ^{k-1})^\mathrm {T}$. (The growth laws of this model family, in addition to von Bertalanffy growth, also encompass two other main growth laws from the literature: logistic, $a(E)x + b(E)x^2$, and Gompertz, $a(E)x -b(E)x \ln (x)$. Just appropriately transform the *x*-axis.) A further extension comes from a mathematical quirk for which we failed to find a biological interpretation: it is possible to add a quadratic term in $\zeta$ to the growth law, $g(x,E) = v_0(x)(v_1(E)+v_2(E)\zeta +v_3(E) \zeta ^2)$, which then should be exactly compensated by a similar additional term in the death rate, $\mu (x,E) = g(x,E)\gamma _0(x) + \mu _0(E)+(k-1)v_3(E)\zeta$. Not only are these terms uninterpretable, the required simultaneous fine tuning of the model components makes them irrelevant in any modelling context coming to mind.
  For this model family $H(E) = -\mu _1 (E)I + DL(E)D^{-1}$ with
  $$\begin{aligned} L(E)=\left( \begin{array}{ccccc} 0 &{}\quad -(k-1)v_3(E) &{}\quad 0 &{}\quad \cdots &{}\quad 0\\ v_1(E) &{}\quad v_2(E) &{}\quad -(k-2)v_3(E) &{}\quad &{}\quad 0 \\ 0 &{}\quad 2v_1(E) &{}2v_2(E) &{}\quad &{}\quad 0\\ \vdots &{}\quad 0 &{}\quad 3v_1(E) &{}\quad &{}\quad 0\\ &{}\quad &{}\quad &{}\quad \ddots &{}\quad \\ 0&{}\quad \cdots &{}\quad \cdots &{}\quad (k-1)v_1(E) &{}\quad (k-1)v_2(E)\\ \end{array}\right) \end{aligned}$$
  with $v_3=0$ in the Daphnia style models.

The matrix *K* occurring in the ODE realising the population input–output relation can be recovered by removing the effect of subtracting the birth operator on both sides of (2.14) to get (2.15): $K(E)=H(E)+ \int _\varOmega \psi _i (\xi )\beta _j(E,d\xi ) C$, with *C* defined by the requirement that $\alpha _j=\sum _h c_{jh}\psi _h$, where the $\alpha _j$ are the weight functions telling how the birth rates depend on the i-states (see Formula (2.15)).

The remainder of the paper is geared to an audience of analysts, and accordingly stresses proofs, instead of interpretation-based heuristics.

### For mathematicians: the context of justification

We shall look at a community as a set of coupled populations. The coupling is mediated by the environment, denoted by *E*. On the one hand individuals react to the environment, on the other hand they influence their environment. We concentrate on a single population and pretend that *E* is a given function of time taking values in a set ${\mathscr {E}}$. So *E* can be regarded as an input. The single population model can serve as a building block for the community model once we have also specified a population level output by additively combining the impact that individuals have.

In order to account for the population dynamical behaviour of individuals (giving birth, dying, impinging on the environment), we first introduce the concept of *individual state* (i-state). Given the course of the environment, the i-states of the individuals independently move through the i-state space and their current position is all that matters at any particular time. The use of the word *state* entails the Markov property; admittedly idealisation is involved and finding the i-state space as well as specifying the relevant environmental variables is often a process of trial and error. The art of modelling comprises deliberate simplification in order to gain significant insights.

We denote the i-state space by $\varOmega$ and assume that it is a subset of ${\mathbb {R}}^n$. In general the *population state* (p-state) is a measure *m* on $\varOmega$ with the interpretation that $m(\omega )$ is the number (per unit of volume or area) of individuals with i-state in the measurable subset $\omega$ of $\varOmega$.

*A word of warning* We usually denote a Borel *subset* of $\varOmega$ by $\omega$. We are aware of a different notational convention in probability theory where $\omega$ denotes a *point* in $\varOmega$. We hope this will not lead to confusion.

In many models the p-state can adequately be represented by a density $n \in L^1(\varOmega )$. The abstract ODE

2.18 $$\begin{aligned} \frac{dn}{dt}(t) = A(E(t))n(t) = A_0(E(t))n(t) + B(E(t))n(t) \end{aligned}$$

captures that the density *n*(*t*) changes in time due to

(i) transport through $\varOmega$ due to i-state development such as growth of individuals, and degradation due to death of individuals,
(ii) reproduction.

The effects of (i) are incorporated in the action of $A_0(E)$ and the effects of (ii) in the action of *B*(*E*). Since the i-states of offspring are, as a rule, quite different from the i-states of the parent, the operator *B*(*E*) is usually non-local. When $x \in \varOmega$ specifies the size of an individual, growth is deterministic and giving birth does not affect the parent’s size, the abstract ODE (2.18) corresponds to the PDE

2.19 $$\begin{aligned} \frac{\partial }{\partial t}n(t,x)= & {} -\frac{\partial }{\partial x}\left( g(x,E(t))n(t,x)\right) -\mu (x,E(t))n(t,x)\nonumber \\&+\int _\varOmega \widetilde{\beta }(y,E(t),x)n(t,y)dy, \end{aligned}$$

with $g,\, \mu$ and $\int _\varOmega \widetilde{\beta }(\cdot ,\cdot ,x)dx$ denoting, respectively, the growth, death, and reproduction rate of an individual with the specified i-state under the specified environmental condition.

The mathematical justification of (2.18) or (2.19) is cumbersome. In particular, it is difficult to give a precise characterisation of the domains of the various unbounded operators. Note that we are not in the setting of (generators of) semigroups of linear operators for which some results can be found in the paper by Atay and Roncoroni (2017). Indeed, we are dealing with evolutionary systems and for this non-autonomous analogue of semigroups a one-to-one correspondence between an evolutionary system and a generating family of differential operators is not part of a well-established theory (but see our earlier work (Clément et al. 1988; Diekmann et al. 1995) for some results in that direction). As shown by Diekmann et al. (1998, 2001), one can actually avoid the differential operators. In the next section we present this approach in the setting where, because of assumptions concerning reproduction, we can work with densities rather than measures. In the present section we simply ignore the mathematical difficulties and proceed formally.

If the test described in Sects. 4.1 and 2.1.4 yields a positive result in the end, it provides

- an integer *k*,
- a bounded linear map $P:L^1(\varOmega ) \rightarrow {\mathbb {R}}^k$,
- a family *K*(*E*) of $k \times k$ matrices,

such that

2.20 $$\begin{aligned} PA(E) =K(E)P \end{aligned}$$

and accordingly

2.21 $$\begin{aligned} N(t):= Pn(t), \end{aligned}$$

where *n*(*t*) is a solution of (2.18), satisfies the ODE

2.22 $$\begin{aligned} \frac{d}{dt}N(t)= K(E(t))N(t). \end{aligned}$$

The p-output that is needed in the community model, which is our ultimate interest, as well as any other p-output that we are interested in, was the starting point for the test and thus is incorporated in *N*, so we can focus our attention on the finite dimensional ODE (2.22) and forget about the infinite dimensional version (2.18) from which it was derived.

A special case occurs when there exist

- a family *H*(*E*) of $k \times k$ matrices,
- a family *Q*(*E*) of bounded linear maps from ${\mathbb {R}}^k$ to $L^1(\varOmega )$

such that

2.23 $$\begin{aligned} PA_0(E)= & {} H(E)P, \end{aligned}$$

2.24 $$\begin{aligned} B(E)= & {} Q(E)P. \end{aligned}$$

(Incidentally, we here took $L^1(\varOmega )$ as the range space for *Q*(*E*), but actually we shall allow *Q*(*E*) to take on values in a linear subspace of the vector space of all Borel measures on $\varOmega$, see Sect. 4.) This case amounts to taking

2.25 $$\begin{aligned} K(E)= H(E) + M(E). \end{aligned}$$

with

2.26 $$\begin{aligned} M(E)=PQ(E). \end{aligned}$$

The key nice features of this special case are:

(i) $A_0(E)$ is a strictly local operator and this allows us to make an in-depth study of the characterisation of those $A_0(E)$ for which *P* and *H*(*E*) exist.
(ii) $B(E)n =Q(E)N$ and hence we can rewrite (2.18) in the form
  2.27 $$\begin{aligned} \frac{dn}{dt} = A_0(E)n +Q(E)N. \end{aligned}$$
  The same local character of $A_0(E)$ now allows us to solve (2.27) explicitly, thus expressing the part of the population that is born after the time at which we put an initial condition explicitly in terms of *N*(*t*). See Proposition 7.2 for a concrete example.

If we ignore birth, that is, set $B(E)=0$ ($\widetilde{\beta }(\cdot ,E,\cdot ) =0$), then the abstract ODE (2.18) and its PDE counterpart (2.19) become transport-degradation equations and we only have to consider condition (2.23). In this paper we give necessary and sufficient conditions in terms of *g* and $\mu$ for the existence of $k,\,P$ and *H*(*E*) such that (2.23) holds and hence *N*(*t*) satisfies (2.22) (with $K(E)=H(E)$, or, equivalently $M(E)=0$). Subsequently we view condition (2.24) as a *restriction* on the submodel for reproduction. If $\widetilde{\beta }$ is such that the restriction (2.24) holds, then the full infinite-dimensional system (2.18) is reducible to the finite dimensional ODE (2.22). In this manner we obtain a catalogue of models that are ODE-reducible and within a restricted class of models with one-dimensional i-state space the catalogue is even complete.

Let $w_i\,\, i=1,2,\ldots ,k$ be bounded measurable functions defined on $\varOmega$ such that

2.28 $$\begin{aligned} \left( Pn\right) _i = \left\langle n,w_i\right\rangle = \int _\varOmega n(x)w_i(x)dx. \end{aligned}$$

Then condition (2.23) amounts to

2.29 $$\begin{aligned} A_0^*(E)w_i = \sum _{j=1}^k H_{ij}(E)w_j. \end{aligned}$$

We might call $A_0^*(E)$ the Kolmogorov backward operator although strictly speaking that operator acts on the continuous functions on $\varOmega$ and is the pre-adjoint of the forward operator acting on the measures on $\varOmega$. Since the elements $w_i$ that figure in our catalogue are continuous functions, the distinction between $A_0^*(E)$ and the Kolmogorov backward operator is inessential.

In words (2.29) states that the *E*-independent subspace spanned by $\{w_1,w_2,\ldots , w_k\}$ is in the domain of $A_0^*(E)$ and invariant under $A_0^*(E)$ for all relevant *E*. To avoid redundancy one should choose *k* as small as possible and we therefore require that the functions $w_i:\varOmega \rightarrow {\mathbb {R}},\, i=1,2,\ldots ,k,$ are linearly independent.

When (2.18) represents (2.19), condition (2.23) with *P* given by (2.28), amounts to

2.30 $$\begin{aligned} g(x,E)w_i'(x)-\mu (x,E)w_i(x) = \sum _{j=1}^k H_{ij}(E)w_j(x),\quad i=1,2,\ldots ,k. \end{aligned}$$

It is easy to find a solution: if

2.31 $$\begin{aligned} \mu (x,E) = \gamma _0(x)g(x,E) + \mu _0(E) \end{aligned}$$

for some functions $\gamma _0:\varOmega \rightarrow {\mathbb {R}}$ and $\mu _0:{\mathscr {E}}\rightarrow {\mathbb {R}}$, then the choice

2.32 $$\begin{aligned} k= & {} 1,\nonumber \\ H(E)= & {} -\mu _0(E),\nonumber \\ w(x)= & {} \exp \left( \int _{x_b}^x \gamma _0(y)dy\right) ,\,\hbox {for some}\,\, x_b \in \varOmega , \end{aligned}$$

makes (2.30) a valid equality. If we restrict to $k=1$, then this is in fact the only possibility.

As mentioned above, condition (2.24) is a restriction on reproduction. The smaller the value of *k*, the more severe is the restriction. We therefore want to make *k* as large as possible while retaining the linear independence of $\{w_1,w_2,\ldots , w_k\}$. So the question arises whether it is possible to pinpoint more restrictive conditions on *g* and $\mu$ that allow for arbitrarily large values of *k*.

If the growth rate of an individual does not depend on its i-state but only on the environmental condition, that is, if

2.33 $$\begin{aligned} g(x,E)=v(E) \end{aligned}$$

for some function $v:\varOmega \rightarrow {\mathbb {R}}$, we call the i-state *physiological age* and often talk about maturation rather than growth. If on top of (2.33) we assume (2.31) and replace the unknown *w* by $\widetilde{w}$ via the transformation

2.34 $$\begin{aligned} w_i(x)= \exp \left( \int _{x_b}^x \gamma _0(y)dy\right) \widetilde{w}_i(x), \quad i=1,2,\ldots ,k, \end{aligned}$$

then (2.30) is equivalent to

2.35 $$\begin{aligned} \widetilde{w}'(x) = \frac{\mu _0(E)}{v(E)}\widetilde{w}(x) + \frac{1}{v(E)}H(E)\widetilde{w}(x). \end{aligned}$$

If we choose

2.36 $$\begin{aligned} H(E) = v(E)\varLambda -\mu _0(E) I, \end{aligned}$$

with $\varLambda$ an arbitrary $k \times k$ matrix for arbitrary *k*, then the *E*-dependence vanishes from Eq. (2.35), which becomes an autonomous linear ODE with solution

2.37 $$\begin{aligned} \widetilde{w}(x) = e^{(x-x_b)\varLambda } \widetilde{w}(x_b). \end{aligned}$$

To avoid redundancy, we have to make sure that $\varLambda$ and $\widetilde{w}(x_b)$ are such that the components of $\widetilde{w}$ are linearly independent as scalar functions of the variable $x\in \varOmega$, see conditions (H1) to (H3) of Sect. 5 as well as Corollary 6.3.

If instead of (2.33) we assume

2.38 $$\begin{aligned} g(x,E)=v_0(x)v_1(E) \end{aligned}$$

for some functions $v_0:\varOmega \rightarrow {\mathbb {R}}$ and $v_1:{\mathscr {E}}\rightarrow {\mathbb {R}}$, we can introduce $\zeta$ defined in terms of *x* by

2.39 $$\begin{aligned} \zeta (x) = \exp \left( \int _{x_b}^x \frac{dy}{v_0(y)}\right) \end{aligned}$$

as a new i-state variable and thus reduce the situation to (2.33).

In Example 1.2 we have

2.40 $$\begin{aligned} g(x,E)=v_1(E) + v_2(E)x. \end{aligned}$$

In combination with (2.31) this leads to

2.41 $$\begin{aligned} \left( v_1(E)+v_2(E)x\right) \widetilde{w}'(x) =H_0(E)\widetilde{w}(x), \end{aligned}$$

where $\widetilde{w}$ is once more defined by (2.34) and where we have put

2.42 $$\begin{aligned} H_0(E) = H(E) + \mu _0(E)I. \end{aligned}$$

For arbitrary *k* we can make (2.41) into an identity by choosing for $i=1,2,\ldots ,k$

2.43 $$\begin{aligned} \widetilde{w}_i(x) = x^{i-1} \end{aligned}$$

and the entries of the matrix $H_0(E)$

2.44 $$\begin{aligned} \left( H_0(E)\right) _{ij} = \left\{ \begin{array}{ll} j v_1(E) &{}\quad \hbox { if}\ j=i-1,\\ (j-1)v_2(E) &{}\quad \hbox { if}\ j=i,\\ 0 &{}\quad \hbox { otherwise.} \end{array} \right. \end{aligned}$$

Again we can allow *g* to have an extra factor $v_0(x)$ since we can remove it by the transformation from *x* to $\zeta$ defined by (2.39).

If instead of (2.40) we assume that

2.45 $$\begin{aligned} g(x,E)=v_1(E) + v_2(E)x + v_3(E)x^2 \end{aligned}$$

we can, as somewhat more complicated computations show, keep the $\widetilde{w}$ specified in (2.43), but adapt $H_0(E)$ slightly as follows:

2.46 $$\begin{aligned} \left( H_0(E)\right) _{ij} = -(k-i)v_3(E) \,\, \hbox { if}\, j=i+1, \end{aligned}$$

while keeping the entries for all other combinations of *i* and *j* as in (2.44). But in addition we need to replace (2.31) by

2.47 $$\begin{aligned} \mu (x,E) = \gamma _0(x)g(x,E) + \mu _0(E) + (k-1)v_3(E)x, \end{aligned}$$

which, *nota bene*, involves *k*. So if we consider *g* and $\mu$ as given, there can be at most one *k* for which this works. Again we can allow a factor $v_0(x)$ in *g* and work with $\zeta$ defined by (2.39).

The main result of the present paper is that problem (2.30), with $\{w_1,w_2,\ldots , w_k\}$ linearly independent, admits no other solution than the ones described above.

## Physiologically structured population models

The formulation of a population model starts at the individual level with the specification of the *individual states* (i-states for short) representing physiological conditions that distinguish individuals from one another. The set of all admissible i-states is denoted by $\varOmega$. In the present paper we restrict ourselves to the case of a finite dimensional $\varOmega \subset {\mathbb {R}}^n$ which we make a measurable space by equipping it with the $\sigma$-algebra $\varSigma$ of all Borel sets. This measurable space is called the *individual state space* (i-state space). Our main results concern $n=1$ with $\varOmega$ an interval, possibly of infinite length. In that case one may think of the i-state as, for example, the size of an individual and we shall indeed often refer to the i-state as size.

The world in which individuals lead their lives has an impact on their development and behaviour. We capture the relevant aspects of the external world in a variable called the *environmental condition* denoted by *E* and taken from a set denoted by ${\mathscr {E}}$. One may think of *E* as a specification of food concentration, predation pressure and, possibly, other quantities like temperature or humidity.

Dependence among individuals arises from a feedback loop: the individuals themselves exert an influence on the environmental condition, for instance, by consuming food or serving as food for predators. As a rule, this feedback loop involves several species. We refer to the paper by Diekmann et al. (2010) for a concrete example. Note, however, that the example of cannibalism shows that this rule is not universal.

We consider the environmental condition as *input* and investigate how the input leads to *output* that comprises the contribution to the (change of) the environmental condition of the species itself, or other species, or any other quantity that we happen to be interested in. By taking population outputs as inputs for other populations or inanimate resources, we can build a dynamical model of a community. The ultimate model incorporates dependence among individuals and leads to nonlinear equations. But each building block considers the environmental condition as a given input and computes population output by summing the contributions by individuals. The present paper focusses on the *population state* (p-state for short) linear (but otherwise nonlinear) input–output map generated by a single building block.

The processes that have to be modelled are:

- i-state development (called *growth* for short),
- survival,
- reproduction (how much offspring and with what i-state at birth).

We assume that, given the environmental condition, growth is deterministic. We further assume that reproduction can be described by a per capita rate. We thus exclude, for instance, cell fission occurring exactly when the mother cell reaches a threshold size (see Example 8.5). Accordingly, and in line with Metz and Diekmann (1986) and de Roos et al. (2013), we introduce the three key model ingredients:

- the *growth rate* *g*(*x*, *E*),
- the *death rate* $\mu (x,E)$,
- the *reproduction rate* $\beta (x, E, \omega ),\, \omega \in \varSigma$.

Again we warn the readers that our use of the symbol $\omega$ for a measurable subset of $\varOmega$ differs from the notational convention in probability theory.

The reproduction rate $\beta$ should be interpreted as follows: the rate at which an individual of size *x* gives birth under environmental condition *E* is $\beta (x, E, \varOmega )$ and the state-at-birth of the offspring is distributed according to the Borel probability measure $\beta (x, E, \cdot )/\beta (x, E, \varOmega )$.

Once we have a model at the i-level, it is a matter of bookkeeping to lift it to the p-level (Metz and Diekmann 1986; Diekmann and Metz 2010): Equating a p-level fraction to an i-level probability one obtains the deterministic (that is, the large population limit) link between the two levels. Still there are choices to be made for the formalism to employ: it could be partial differential equations (Metz and Diekmann 1986; Perthame 2007; Gwiazda and Marciniak-Czochra 2010) or renewal equations (RE) (Diekmann et al. 1998, 2001). Here we choose RE, albeit not in the most general form, since reproduction is described by a per capita rate.

As a first step we build composite ingredients from the basic ingredients $g, \mu$, and $\beta$. We shall do so without specifying the nature of the environmental condition *E*, in particular, without specifying the space ${\mathscr {E}}$ to which *E* belongs. Often we conceive of the environmental condition as a given function of time. When *E* occurs as a subscript to a function with argument (*t*, *s*) this entails that $E(\tau )$ is given for $\tau \in [s,t]$.

We shall provide a constructive definition of the following quantities:

$$\begin{aligned} X_E(t,s,x):= & {} \hbox {the i-state at time }t,\hbox { given survival and given the input }\\&\tau \mapsto E(\tau ),\hbox { of an individual that had i-state }x \in \varOmega \hbox { at time }\\&s \le t, \\ {\mathscr {F}}_E(t,s,x):= & {} \hbox { the survival probability up to time }t,\hbox { given the input }\tau \mapsto E(\tau ),\\&\hbox { of an individual that had i-state }x \in \varOmega \hbox { at time }\\&s \le t. \end{aligned}$$

We *assume* that *g* and $\tau \mapsto E(\tau )$ are such that the initial value problem

3.1 $$\begin{aligned} \frac{d\xi }{d\tau }(\tau ) = g(\xi (\tau ), E(\tau )), \quad \xi (s) =x \end{aligned}$$

has a unique solution $\xi (\tau )$ on [*s*, *t*] and define

3.2 $$\begin{aligned} X_E(t,s,x):= \xi (t). \end{aligned}$$

We further *assume* that $\mu ,\, g$, and $\tau \mapsto E(\tau )$ are such that also the initial value problem

3.3 $$\begin{aligned} \frac{df}{d\tau }(\tau ) = -\mu (\xi (\tau ), E(\tau ))f(\tau ), \quad f(s) =1 \end{aligned}$$

has a unique solution $f(\tau )$ on [*s*, *t*] and define

3.4 $$\begin{aligned} {\mathscr {F}}_E(t,s,x):= f(t). \end{aligned}$$

Let

$$\begin{aligned} u^0_E(t,s,x,\omega ):= & {} \hbox { probability that, given the input }\tau \mapsto E(\tau ),\\&\hbox { an individual that had i-state }\\&x \in \varOmega \hbox { at time }s \le t\hbox { is still alive at time }\\&t\hbox { and has i-state in the set }\omega \in \varSigma . \end{aligned}$$

Then, since the growth of an individual is deterministic, we have

3.5 $$\begin{aligned} u^0_E(t,s,x,\omega ) = {\mathscr {F}}_E(t,s,x) \delta _{X_E(t,s,x)}(\omega ), \end{aligned}$$

that is, the (unless survival is guaranteed) defective probability distribution $u^0_E(t,s,x,\cdot )$ is a point measure concentrated at position $X_E(t,s,x) \in \varOmega$ of mass ${\mathscr {F}}_E(t,s,x)$. Let

$$\begin{aligned} \beta ^1_E(t,s,x,\omega ):= & {} \hbox { the rate at which, given the input }\tau \mapsto E(\tau ),\\&\hbox { an individual that had i-state }\\&x \in \varOmega \hbox { at time }s \le t\hbox { produces at time }t\\&\hbox { offspring with i-state-at-birth in the set }\omega \in \varSigma . \end{aligned}$$

Then

3.6 $$\begin{aligned} \beta ^1_E(t,s,x,\omega ) = {\mathscr {F}}_E(t,s,x)\beta \left( X_E(t,s,x),E(t),\omega \right) . \end{aligned}$$

### Lemma 3.1

Given the input $\tau \mapsto E(\tau )$ defined on [*s*, *t*], the relations

3.7 $$\begin{aligned} u^0_E(t,s,x,\omega )= & {} \int _\varOmega u^0_E(t,\tau ,y,\omega ) u^0_E(\tau ,s,x,dy), \end{aligned}$$

3.8 $$\begin{aligned} \beta ^1_E(t,s,x,\omega )= & {} \int _\varOmega \beta ^1_E(t,\tau ,y,\omega ) u^0_E(\tau ,s,x,dy)\nonumber \\= & {} {\mathscr {F}}_E(\tau ,s,x) \beta ^1_E\left( t,\tau ,X_E(\tau ,s,x),\omega \right) \end{aligned}$$

hold for all $\tau \in (s,t)$ and all $\omega \in \varSigma$.

Relation (3.7) is the Chapman–Kolmogorov equation and (3.8) is a similar consistency relation relating growth, survival and reproduction.

We omit the straightforward proof of Lemma 3.1, but note that, essentially, the consistency relations reflect the uniqueness of solutions to Eqs. (3.1) and (3.3).

The composite ingredients $u^0_E$ and $\beta ^1_E$ satisfying (3.7) and (3.8) are the starting point for a next round of constructive definitions. They are examples of *kernels parametrised by the input* (cf. Diekmann et al. 2001). Such a kernel $\phi _E$ assigns to each input $\tau \mapsto E(\tau )$ defined on [*s*, *t*] a function $\phi _E(t,s,\cdot ,\cdot ): \varOmega \times \varSigma \rightarrow {\mathbb {R}}$ which is bounded and measurable with respect to the first variable and countably additive with respect to the second variable. This is to say that for fixed $\omega \in \varSigma$ the function $x \mapsto \phi _E(t,s,x,\omega )$ is bounded and measurable while for fixed $x \in \varOmega$ the map $\omega \mapsto \phi _E(t,s,x,\omega )$ is a finite signed measure.

The *product* $(\phi \star \psi )_E$ of two kernels $\phi _E$ and $\psi _E$ parametrised by the input is defined by

3.9 $$\begin{aligned} (\phi \star \psi )_E(t,s,x,\omega ) = \int _s^t \int _\varOmega \phi _E(t,\tau ,y,\omega )\psi _E(\tau ,s,x,dy)d\tau . \end{aligned}$$

The $\star$-product is associative.

For $k \ge 2$ we define recursively

3.10 $$\begin{aligned} \beta _E^k := \left( \beta ^1 \star \beta ^{k-1}\right) _E \end{aligned}$$

The interpretation of $\beta _E^k$ is as follows: Given the input $\tau \mapsto E(\tau )$ defined on [*s*, *t*], the quantity $\beta _E^2(t,s,x,\omega )$ is the rate at which grandchildren to an individual that had i-state *x* at time *s* are born at time *t* with i-state-at-birth in the set $\omega \in \varSigma$. The quantity $\beta _E^3(t,s,x,\omega )$ has the same interpretation but for great-grandchildren and $\beta _E^k(t,s,x,\omega )$ for *k*th generation offspring. To get the combined birth rate of all descendants of such an individual we sum up over all generations and define

3.11 $$\begin{aligned} \beta _E^c := \sum _{k=1}^\infty \beta _E^k, \end{aligned}$$

where the superscript *c* stands for *clan*.

Because every member of the clan is either a child of the ancestor or a child of a member of the clan, or, alternatively, either a child of the ancestor or a member of the clan of a child of the ancestor, we obtain a consistency relation in terms of the following RE:

3.12 $$\begin{aligned} \beta _E^c = \beta _E^1 + \left( \beta ^1 \star \beta ^c\right) _E = \beta _E^1 + \left( \beta ^c \star \beta ^1\right) _E. \end{aligned}$$

Mathematically, (3.12) means that $\beta _E^c$ is the *resolvent kernel* of the kernel $\beta _E^1$ (cf. Gripenberg et al. 1990).

In order to incorporate both the founding ancestor and the development of the descendants after birth, we finally define

3.13 $$\begin{aligned} u_E^c =u_E^0 + \left( u^0 \star \beta ^c\right) _E. \end{aligned}$$

For later use we note that

3.14 $$\begin{aligned} \beta _E^c(s,s,x,\omega ) = \beta _E^1(s,s,x,\omega ) = \beta (x,E(s),\omega ) \end{aligned}$$

and

3.15 $$\begin{aligned} u_E^c(s,s,x,\omega ) = u_E^0(s,s,x,\omega ) = \delta _x(\omega ). \end{aligned}$$

The clan-kernels $u_E^c$ and $\beta _E^c$ satisfy the same consistency relations as $u_E^0$ and $\beta _E^1$ of Lemma 3.1.

### Theorem 3.2

Given the input $\tau \mapsto E(\tau )$ defined on [*s*, *t*], the relations

3.16 $$\begin{aligned} u^c_E(t,s,x,\omega )= & {} \int _\varOmega u^c_E(t,\tau ,y,\omega ) u^c_E(\tau ,s,x,dy), \end{aligned}$$

3.17 $$\begin{aligned} \beta ^c_E(t,s,x,\omega )= & {} \int _\varOmega \beta ^c_E(t,\tau ,y,\omega ) u^c_E(\tau ,s,x,dy) \end{aligned}$$

hold for all $\tau \in (s,t)$ and all $\omega \in \varSigma$.

The proof of Theorem 3.2 proceeds by proving (3.17) first and next use this identity to verify (3.16). The papers (Diekmann et al. 1998, 2001, 2003) contain a much more detailed exposition of this constructive approach, including proofs of (3.16) and (3.17) in a generalised form with the instantaneous rate $\beta _E^1$ replaced by cumulative offspring production $\varLambda$, necessitating the use of the Stieltjes integral, which can be avoided here since we assume that offspring are produced at a per capita rate, so with some probability per unit of time. In our paper (Diekmann et al. 1998) we considered general linear time dependent problems (the time dependence corresponding to fixing an input). In the paper (Diekmann et al. 2001) we explicitly considered input, but focussed on the feedback loop that captures dependence. As the notation of (Diekmann et al. 2001) is not ideal for investigating the problem introduced in the next section, we have adopted in the present exposition a different, more suitable notation, viz. the use of the subscript *E*. In the paper (Diekmann et al. 2003) the main objective was to characterise the steady states.

The central idea of the modelling and analysis methodology of physiologically structured populations is that we view the population state as a measure *m* on $\varOmega$. We use the kernel $u_E^c$ to define operators $U_E^c(t,s)$ that map the p-state at time *s* to the p-state at time *t* as follows. Assuming that all individuals experience the same environmental condition *E*, we can associate to each measure *m* a new measure

3.18 $$\begin{aligned} \left( U_E^c(t,s)m\right) (\omega ) := \int _\varOmega u_E^c(t,s,x,\omega ) m(dx) \end{aligned}$$

and note that the Chapman–Kolmogorov relation (3.16) translates into the semigroup property

3.19 $$\begin{aligned} U_E^c(t,s) = U_E^c(t,\tau )U_E^c(\tau ,s), \quad s<\tau <t, \end{aligned}$$

while (3.15) yields

3.20 $$\begin{aligned} U_E^c(s,s) =I. \end{aligned}$$

Note that we may replace the superscript *c* in (3.18) by 0 and use (3.7) to deduce the semigroup property for $U_E^0$. That $U_E^0(s,s)=I$ follows again from (3.15). Families of linear maps that satisfy the conditions (3.18) and (3.20) are called *state-linear dynamical systems with input*.

In a previous paper (Diekmann et al. 2018) we have already considered what amounts to population level conditions for ODE-reducibility, in this paper we concentrate on finding i-level ones.

## Finite dimensional state representation

### General considerations

Let *Y* be a vector space. We do not yet fix a topology for *Y*, but note that if $Y'$ is a separating vector space of linear functionals on *Y*, then the weakest topology on *Y* for which all $y' \in Y'$ are continuous (the so-called $w(Y,Y')$-topology) makes *Y* into a locally convex space whose dual space is $Y'$ (Rudin 1973; Theorem 3.10, p.62).

Let $U_E$ be a state-linear dynamical system with input $\tau \mapsto E(\tau )$, which at this point has no connection yet with either $U^0_E$ or $U^c_E$. This means that each $U_E(t,s)$ is a linear operator on *Y* and

4.1 $$\begin{aligned} U_E(s,s)= & {} I, \end{aligned}$$

4.2 $$\begin{aligned} U_E(t,s)= & {} U_E(t,\tau )U_E(\tau ,s), \quad s< \tau < t. \end{aligned}$$

If a vector topology has been chosen for *Y* we also require the operators $U_E(t,s)$ to be continuous with respect to this topology.

We are interested in finding a finite dimensional exact reduction (or, lumping) of $U_E(t,s)$. More precisely, we want, if possible, to choose a separating vector space $Y'$ of linear functionals on *Y* and construct a $w(Y,Y')$-continuous linear map $P: Y \rightarrow {\mathbb {R}}^k$ and a $k \times k$ matrix *K*(*E*) such that

4.3 $$\begin{aligned} PU_E(t,s) = \varPhi _E(t,s)P, \end{aligned}$$

where $\varPhi _E(t,s)$ is the fundamental matrix solution of the *k*-dimensional ODE-system

4.4 $$\begin{aligned} \frac{dN}{d\tau }(\tau ) = K(E(\tau ))N(\tau ). \end{aligned}$$

The $w(Y,Y')$-continuous linear map *P* can be represented by elements $W_i \in Y',\, i=1,2,\ldots ,k$ as

4.5 $$\begin{aligned} \left( Py\right) _i = \left\langle y,W_i\right\rangle . \end{aligned}$$

To avoid useless variables, *P* should be surjective or, equivalently, the functionals $W_1, W_2,\ldots , W_k$ should be linearly independent.

The adjoint of a forward evolutionary system characterised by (4.1)–(4.2) is a backward evolutionary system (see Clément et al. 1988; Diekmann et al. 1995). For these it is more natural to think of *s* as the dynamic variable with respect to which we differentiate. Since the restriction of an evolutionary system to the diagonal in the (*t*, *s*)-plane is the identity operator, the derivative with respect to *t* is simply minus the derivative with respect to *s* at the diagonal. This observation suffices for our purpose and we therefore do not elaborate the forward-backward duality here.

Let *W* denote the *k*-vector with components $W_i$. We may then rewrite (4.3) in the form

4.6 $$\begin{aligned} \left( U_E(t,s)\right) ^*W = \varPhi _E(t,s)W \end{aligned}$$

as a shorthand for

4.7 $$\begin{aligned} \left( U_E(t,s)\right) ^*W_i = \sum _{j=1}^k \left( \varPhi _E(t,s)\right) _{ij}W_j, \quad i=1,2,\ldots ,k. \end{aligned}$$

Since the right hand side of (4.7) is differentiable, the same must be true for the left hand side. By differentiation we obtain the following task:

$$\begin{aligned} \hbox {TASK}_{\mathrm{L}}{:} \quad \frac{d}{dt} \left( U_E(t,s)\right) ^*W_{\big | t=s} = K(E(s))W. \end{aligned}$$

Here the subscript L refers to *lumpability* and the task is to find *k* elements $W_i \in Y^*$ such that

- the derivatives exist,
- the outcome is a linear combination, with input dependent coefficients, of the elements $W_i$.

Assuming that the derivative exists, we may write

4.8 $$\begin{aligned} \frac{d}{dt} \left( U_E(t,s)\right) ^*W_{\big | t=s} = A(E(s))^*W \end{aligned}$$

and in Sects. 2.1 and 8 we employ the notation of the right hand side.

In this paper we accomplish $\hbox {TASK}_{\mathrm{L}}$ for the state-linear dynamical system $U_E^0$ with input introduced in Sect. 3. In fact, we characterise the growth rates *g* and the death rates $\mu$ for which exact reduction is possible and we compute the corresponding *W* and *K*(*E*). In order to explain the relevance of these results for the dynamical system $U_E^c$ we now widen the perspective by introducing output.

**Fig. 2:**
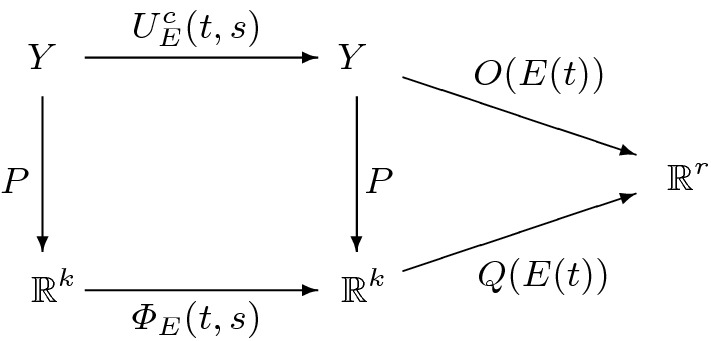
Structure of models with output and finite dimensional reduction

We return to (4.1) and (4.2) and complement them by a $w(Y,Y')$-continuous linear map

4.9 $$\begin{aligned} O(E): Y \rightarrow {\mathbb {R}}^r. \end{aligned}$$

We call

4.10 $$\begin{aligned} O(E(t))U_E(t,s)y \end{aligned}$$

the *output* at time *t*, given the state *y* at time *s* and the input $\tau \mapsto E(\tau )$ defined on [*s*, *t*]. The idea is that the state itself is not observable, only the output can be measured. We now ask the following question: can, in fact, the relation between the pair (initial state, input) on the one hand and output on the other, alternatively and equivalently be described in terms of a *finite dimensional* dynamical system? That is, when is the diagram in Fig. 2 commutative for all inputs $\tau \mapsto E(\tau )$ defined on [*s*, *t*]?

More precisely, we again want to find an integer *k* and continuous linear maps *P* and *K*(*E*), but in addition to (4.3) we now require

4.11 $$\begin{aligned} O(E(t)) U_E(t,s) =Q(E(t)) \varPhi _E(t,s)P, \end{aligned}$$

where the $k \times r$ matrix *Q*(*E*) is also to be determined. In other words, we require that, given the state *y* at time *s* and the input $\tau \mapsto E(\tau )$ defined on [*s*, *t*], the output at time *t* is obtained by applying *Q*(*E*(*t*)) to the solution at time *t* of (4.4) with initial condition

4.12 $$\begin{aligned} N(s)=Py. \end{aligned}$$

We stress that, as far as the output is concerned, the reduction does *not* involve loss of information. At the black box level we cannot distinguish between the true system $U_E$ and its finite dimensional counterpart $\varPhi _E$.

#### Remark 4.1

In principle we could allow *P* to depend on *E* and write the initial condition (4.12) as

4.13 $$\begin{aligned} N(s)=P(E(s))y. \end{aligned}$$

But if we can choose *E*(*s*) without affecting $U_E(t,s)$, then, since also $\varPhi _E(t,s)$ is insensitive to the precise value of *E* in *s*, it follows that after all, *P* cannot depend on *E*. A similar observation was made for a related problem in Remark 7.3 of (Diekmann et al. 2018).

Taking $t=s$ in (4.11) we find that necessarily

4.14 $$\begin{aligned} O(E)=Q(E)P. \end{aligned}$$

This allows us to rewrite (4.11) as

4.15 $$\begin{aligned} Q(E(t)) \left( PU_E(t,s) - \varPhi _E(t,s)P\right) = 0. \end{aligned}$$

The aim is now to derive necessary and sufficient conditions on *O*(*E*) and $U_E$ for the existence of $P,\, Q(E),\, K(E)$ that make (4.15) a valid identity for a *minimal value* of *k*. Note that for $k>r$ the Eq. (4.15) allows for possibly redundant information in (4.4). Indeed, adding components to *N* that are unobservable, in the sense that they do not contribute directly or indirectly to future output, does not harm; by requiring *k* to be minimal we avoid such redundancy. To derive the conditions, we follow an iterative procedure that is well-known in systems theory.

Our starting point is the output map *O*(*E*). For (4.14) to be possible at all, the range of $O(E)^*: {\mathbb {R}}^r \rightarrow Y'$ should be contained in a finite dimensional subspace which is independent of *E*. Without loss of generality we can assume that this subspace has dimension *r*. Indeed, if the dimension is less than *r* there is dependence among the output components and by choosing suitable coordinates we can reduce *r* without losing any information.

Let $\left\{ W_1^0, W_2^0,\ldots , W_r^0\right\} \subset Y'$ be a basis for the subspace of $Y'$ that contains the range of $O(E)^*$. Recall the representation (4.5) of *P* in terms of the elements $W_i \in Y',\, i=1,2,\ldots ,k$. From (4.14) we conclude that for all $i=1,2,\ldots ,r$, the element $W_i^0$ belongs to the subspace spanned by $\left\{ W_1, W_2,\ldots , W_k\right\}$. In particular, $k \ge r$. By a suitable choice of basis for ${\mathbb {R}}^k$ we can arrange things so that

4.16 $$\begin{aligned} W_i = W_i^0,\quad i=1,2,\ldots , r. \end{aligned}$$

Define $$P_0:Y \rightarrow {\mathbb {R}}^r$$ by

4.17 $$\begin{aligned} \left( P_0y\right) _i = \left\langle y,W_i^0\right\rangle ,\quad i=1,2,\ldots , r. \end{aligned}$$

Then

4.18 $$\begin{aligned} O(E)=Q_0(E)P_0 \end{aligned}$$

with $Q_0(E): {\mathbb {R}}^r \rightarrow {\mathbb {R}}^r$ such that for all $v \in {\mathbb {R}}^r$

4.19 $$\begin{aligned} O(E)v =0 \,\, \hbox { for all} \,\, E \in {\mathscr {E}}\quad \Rightarrow \quad v=0 \end{aligned}$$

because we have chosen *r* in the optimal way and $\left\{ W_1^0, W_2^0,\ldots , W_r^0\right\}$ is a basis for the range of $O(E)^*$. In the first step in the iterative procedure we try to find $K_0(E):{\mathbb {R}}^r \rightarrow {\mathbb {R}}^r$ such that (4.15) in the guise of

4.20 $$\begin{aligned} Q_0(E(t)) \left( P_0U_E(t,s) - \varPhi _E(t,s)P_0\right) = 0. \end{aligned}$$

holds, where $\varPhi _E(t,s)$ is now the fundamental matrix solution of the *r*-dimensional system

4.21 $$\begin{aligned} \frac{dN}{d\tau }(\tau ) = K_0(E(\tau ))N(\tau ). \end{aligned}$$

On account of (4.19) we may reduce (4.20) to

4.22 $$\begin{aligned} P_0U_E(t,s) = \varPhi _E(t,s)P_0 \end{aligned}$$

if, as we assume, we can manipulate *E*(*t*) without changing $P_0U_E(t,s)$.

One should compare (4.22) with (4.3), but there is an important difference: determining the map *P* or, equivalently, the elements $W_i, \, i= 1,2,\ldots ,k$ of $Y'$ is part of the task whereas the elements $W_i^0, \, i= 1,2,\ldots , r$ are known because *O*(*E*) is given. So rather than a task, we now have the following test:

$$\begin{aligned} \hbox {TEST}_0:\quad \frac{d}{dt} (U_E(t,s))^*W^0_{\,\,\,\big | t=s} \,\,{\mathop {=}\limits ^{?}}\,\, K_0(E(s))W^0. \end{aligned}$$

In more detail the test consists in answering the following questions:

- Does $\frac{d}{dt}U_E^*(t,s)W_i^0{}_{\big | t=s}$ exist for $i=1,2,\ldots ,r$?
- Is the outcome a linear combination, with input dependent coefficients, of the elements $W_1^0,W_2^0,\ldots , W_r^0$?

If, for some index *i*, the derivative does not exist, finite dimensional state representation is not possible. In contrast, there is still hope that a finite dimensional state representation might be possible if a derivative is not in the span of $\left\{ W_1^0, W_2^0,\ldots , W_r^0\right\}$. If that is the case, we add the derivative to the basis and thus enlarge the subspace. Varying both the index *i* and the value of *E*(*s*), we obtain a new subspace of $Y'$ that may, or may not, be finite dimensional. If it is finite dimensional we perform $\hbox {TEST}_1$ which is the analogue of $\hbox {TEST}_0$, but with $W^0$ extended to $W^1$, a vector the components of which span the new subspace. If necessary this procedure can be repeated. If the process leads after a finite number of steps to a finite dimensional subspace, we are in business. If the process does not terminate, finite dimensional state representation is not possible.

In general, finding *P* and *K*(*E*) such that (4.3) holds, or, in other words, performing $\hbox {TASK}_{\mathrm{L}}$, is hard since, literally, one does not know how to start. In contrast, for a given output *O*(*E*), the tests $\hbox {TEST}_0$, $\hbox {TEST}_1, \ldots$ yield a constructive procedure.

If one does manage to characterise *P* and *K*(*E*) such that (4.3) holds, one can give the output problem a twist: (4.11) holds for all outputs of the form (4.14). Below we shall follow this road while considering reproduction as part of the output. In this manner we can focus on (4.3) for $U_E^0$ and in the end still obtain results for $U_E^c$ as described in the next subsection.

### Physiologically structured population models

As explained in Sect. 3, it is natural to consider the p-state of a physiologically structured population as a measure and therefore the p-state space should be a linear subspace *Y* of the vector space $M(\varOmega )$ of all Borel measures on $\varOmega$. One reason for not choosing the whole $M(\varOmega )$ as p-state space is that we may want to keep biologically relevant quantities such as the total biomass finite. If in the one-dimensional i-state space case *x* denotes the size of an individual, then $\int _\varOmega x m(dx)$ represents the total biomass and it is finite only for measures *m* in a proper subspace of $M(\varOmega )$ if $\varOmega$ is an infinite interval. An other reason is that when we check whether a reduction of the infinite dimensional model is ODE-reducible we construct a $w(Y,Y')$-continuous linear map $P:Y \rightarrow {\mathbb {R}}^k$ or, equivalently, linear functionals $W_i \in Y'$, now representable by locally bounded measurable functions $w_i$ on $\varOmega$ via the pairing

4.23 $$\begin{aligned} (Pm)_i = \left\langle m,W_i\right\rangle = \int _\varOmega w_i(x) m(dx), \end{aligned}$$

and we may end up with functions $w_i$ for which the integral in (4.23) is not finite for every $m \in M(\varOmega )$. So we want these functions $w_i$ to represent elements in $Y'$ and consequently have to restrict *Y* to a suitably chosen subspace of $M(\varOmega )$. The freedom we have in choosing *Y* and $Y'$ therefore comes in very handy.

We denote the ${\mathbb {R}}^k$-valued function with components $w_i$ by *w*. Later we shall show that when a finite dimensional state representation exists for the physiologically structured population model, the function *w* is actually continuous, but not necessarily bounded, on $\varOmega$.

Consider the dynamical systems $U_E^0$ and $U_E^c$ with input of Sect. 3. The system $U_E^0$ represents a transport-degradation process (without reproduction) while $U_E^c$ represents a physiologically structured population model with reproduction.

Using (3.18), in its superscript 0 version, (3.13) and (3.5) we find that for the transport-degradation case, (4.3) amounts to

4.24 $$\begin{aligned} {\mathscr {F}}_E(t,s,x)w(X_E(t,s,x)) =\varPhi _E(t,s)w(x), \end{aligned}$$

while for complete physiologically structured population models, it amounts to

4.25 $$\begin{aligned}&{\mathscr {F}}_E(t,s,x)w(X_E(t,s,x)) \nonumber \\&\qquad + \int _s^t \int _\varOmega {\mathscr {F}}_E(t,\tau ,y)w(X_E(t,\tau ,y)){\mathscr {F}}_E(\tau ,s,x)\beta _E^c(\tau ,s,x,dy)d\tau \nonumber \\&\quad =\varPhi _E(t,s)w(x). \end{aligned}$$

Using in addition (3.14) and (3.5) we can elaborate $\hbox {TASK}_{\mathrm{L}}$ by taking the derivative with respect to *t* of both sides of (4.25) and then putting $t=s$. Since $t \mapsto X_E(t,s,x)$ is differentiable, the differentiability of the left hand side of (4.25) implies that the function $x \mapsto w(x)$ is differentiable, at least in certain directions. More precisely, for $x\in \varOmega$ the linear approximation of $w(x+h)-w(x)$ exists as a map acting on the space of vectors *h* spanned by $g(x,\cdot )$. This map we call (*Dw*)(*x*). We therefore find that necessarily

4.26 $$\begin{aligned} (Dw)(x) g(x,E) -\mu (x,E)w(x) = K(E)w(x). \end{aligned}$$

for the transport-degradation model, and

4.27 $$\begin{aligned} (Dw)(x) g(x,E) -\mu (x,E)w(x) + \int _\varOmega w(y)\beta (x,E,dy) = K(E)w(x). \end{aligned}$$

for the population model.

If the transport-degradation model is ODE-reducible, that is, if we can find a positive integer *k*, linearly independent measurable functions $\{w_1,w_2,\ldots ,w_k\}$ and a $k \times k$ matrix *K*(*E*) so that (4.26) holds, the physiologically structured population model is also ODE-reducible if we impose the following restriction on the reproduction process: There exists a $k \times k$ matrix *M*(*E*) such that

4.28 $$\begin{aligned} \int _{\varOmega } w(y) \beta (x,E,dy) = M(E)w(x). \end{aligned}$$

The reason is simply that when this is the case, we can write the Eqs. (4.26) and (4.27) in a unified way as

4.29 $$\begin{aligned} (Dw)(x) g(x,E)= \left( H(E) + \mu (x,E)I\right) w(x), \end{aligned}$$

where $H(E)=K(E)$ for the transport-degradation model and $H(E)=K(E)-M(E)$ for the population model.

Note that the restriction (4.28) is satisfied if reproduction is (part of the) output in the following sense:

4.30 $$\begin{aligned} \beta (x,E,\cdot ) = \sum _{j=1}^k \beta _j(E,\cdot ) w_j(x), \end{aligned}$$

because then we can take *M*(*E*) to be the $k \times k$ matrix with entries

4.31 $$\begin{aligned} M_{ij}(E) =\int _{\varOmega } w_i(y) \beta _j(E,dy). \end{aligned}$$

But there are other situations in which (4.28) is satisfied. The simplest case is when $M(E)=0$. Note that this does not imply the absence of reproduction, merely that $\beta$ is annihilated by all $w_i$ or, in other words, that the functions $w_i$ measure properties of individuals that are preserved under reproduction (as a concrete example, think of mass in cell-fission models).

Yet another case, viz. the one in which $\beta$ has the form

4.32 $$\begin{aligned} \beta (x,E,\omega ) = \widetilde{\beta }(x,E)\beta _0(\omega ), \end{aligned}$$

will be briefly discussed in Sect. 8.

When $n=1$, that is, when the i-state space is one-dimensional, (4.29) reduces to the following equation:

4.33 $$\begin{aligned} g(x,E)w'(x) = (H(E)+\mu (x,E)I)w(x). \end{aligned}$$

In Sect. 5 we address the following problem: list necessary and sufficient conditions on *g* and $\mu$ for the existence of a measurable function $w: \varOmega \rightarrow {\mathbb {R}}^k$ with a priori unknown *k* such that there exists a $k \times k$ matrix *H*(*E*) for which (4.33) holds.

To solve the problem, we heavily use that (4.33) is a local equation in the *x*-variable. For a fixed value $E_0$ the solution *w* of (4.33) is, essentially, given by a matrix exponential. Once *w* has been determined, we can view (4.33) as a constraint for the ways in which $g,\, \mu$ and *H* can depend on *E*.

## A catalogue of models that admit a finite dimensional state representation

In this section we present an explicit catalogue of all possible combinations of model ingredients that allow a finite dimensional state representation for transport-degradation models with $\varOmega \subset {\mathbb {R}}^1$ an interval that may have infinite length. The catalogue extends naturally to all physiologically structured population models in which the submodel for reproduction is restricted by (4.28).

One should choose the individual state space $\varOmega$ such that every point in it is reachable. The following assumption guarantees this. We shall also make use of it in the proofs of our results.

### Assumption 5.1

There exists a constant environmental condition $E_0$ such that

$$\begin{aligned} g(x,E_0) > 0 \quad \hbox { for all}\,\, x \in \varOmega . \end{aligned}$$

The catalogue consists of three families $F_i,\,\,i=1,2,3,$ of functions *g* and $\mu$ for which we specify *w* and *H*(*E*) such that (4.33) holds. These families involve infinite dimensional parameters in the form of functions $\gamma _0:\varOmega \rightarrow {\mathbb {R}}, \,\, \mu _0:{\mathscr {E}}\rightarrow {\mathbb {R}},\,\, v_0:\varOmega \rightarrow {\mathbb {R}},\,\, v_i:{\mathscr {E}}\rightarrow {\mathbb {R}}, \,\, i=1,2,3$. We do not claim that $g, \,\, \mu$ and *w* are biologically meaningful for all choices of these parameters (in fact they are not). The catalogue simply provides a precise description of the constraints on *g* and $\mu$ that enable an equivalent, as far as the output map defined by *w* is concerned, finite dimensional representation of the corresponding transport-degradation model.

A transformation of the i-state variable affects the death rate $\mu$ and the output function *w* in the usual manner. But since the growth rate needs to keep its interpretation, we have to incorporate a transformation specific factor. If

5.1 $$\begin{aligned} y =\phi (x) \end{aligned}$$

and

5.2 $$\begin{aligned} \frac{dx}{da} = g(x,E), \end{aligned}$$

then

5.3 $$\begin{aligned} \frac{dy}{da}= & {} \phi '(x) \frac{dx}{da} =\phi '(x)g(x,E)=\phi '\left( \phi ^{-1}(y)\right) g\left( \phi ^{-1}(y),E\right) \nonumber \\= & {} \frac{1}{\left( \phi ^{-1}\right) '(y)}g\left( \phi ^{-1}(y),E\right) \end{aligned}$$

and accordingly

5.4 $$\begin{aligned} \widetilde{g}(y,E) = \frac{1}{\left( \phi ^{-1}\right) '(y)}g\left( \phi ^{-1}(y),E\right) \end{aligned}$$

is the growth rate of the transformed i-state variable *y*.

Transformation of the i-state-variable induces a transformation of the parameters in our families $F_i$. As we shall indicate below, the multiplicative factor highlighted by (5.4) allows us to transform the i-state variable in such a way that the formula for the growth rate becomes relatively simple.

Let $L:{\mathbb {R}}^k \rightarrow {\mathbb {R}}^k$ be linear and invertible. If (4.33) holds and we define

5.5 $$\begin{aligned} \widetilde{w}= & {} Lw, \nonumber \\ \widetilde{H}= & {} LHL^{-1}, \end{aligned}$$

then, by applying *L* to the identity (4.33), we obtain

5.6 $$\begin{aligned} g(x,E)\widetilde{w}'(x) = \left( \widetilde{H}(E) +\mu (x,E)I\right) \widetilde{w}(x). \end{aligned}$$

So, if *g* and $\mu$ are given and (*w*, *H*) is a solution to (4.33), then $(\widetilde{w},\widetilde{H})$ defined by (5.5) is also a solution. As (5.5) defines an equivalence relation we see that solutions (*w*, *H*) to (4.33) occur in equivalence classes. In our catalogue we choose *w* such that *H* has a relatively simple form, but one should keep in mind that this choice yields a representative of an equivalence class.

As a reference for integration we choose a reference point $x_b \in \varOmega$. If all individuals are born with the same i-state at birth, we choose this i-state as $x_b$.

We are now ready to present our first family in the catalogue.

**Table:** 

$F_1$: scalar representations	
*k*	$k=1$
Parameters	$\gamma _0:\varOmega \rightarrow {\mathbb {R}},\,\, \mu _0:{\mathscr {E}}\rightarrow {\mathbb {R}}$
*g*	No restriction
$\mu$	$\mu (x,E) = \gamma _0(x)g(x,E) + \mu _0(E)$
*w*	$w(x) = \exp \left( \int _{x_b}^x \gamma _0(y)dy\right)$
*H*	$H(E)=-\mu _0(E)$

The proof that (4.33) is an identity if $F_1$ applies is straightforward and omitted.

In the rest of this section we assume that $k \ge 2$ and we supplement (4.33) with the non-degeneracy condition

1. $w_1,w_2,\ldots ,w_k$ are linearly independent as scalar functions of $x \in \varOmega$.

Among the parameters of the second family are a constant (that is, independent of both *x* and *E*) $k \times k$ matrix $\varLambda$ and a constant vector $w(x_b)\in {\mathbb {R}}^k$ specifying the value of *w* in $x_b$. The identity (4.33) holds for all choices of $\varLambda$ and $w(x_b)$. But (H1) has also to be satisfied. Therefore we have to impose the condition

(H2) the eigenvalues of $\varLambda$ have geometric multiplicity one

on the matrix $\varLambda$ and the condition

(H3) if $\{\theta _1,\theta _2,\ldots ,\theta _k\}$ is a basis for ${\mathbb {R}}^k$ consisting of eigenvectors and generalised eigenvectors of $\varLambda$ and
  $$\begin{aligned} w(x_b) = \sum _{j=1}^k d_j\theta _j, \end{aligned}$$
  then $d_j \ne 0$ whenever $\theta _j$ is a generalised eigenvector of highest rank

on the combination of $\varLambda$ and $w(x_b)$.

We are now ready for the second family in our catalogue.

**Table:** 

$F_2$: physiological age	
*k*	$k \ge 2$
Parameters	$\gamma _0:\varOmega \rightarrow {\mathbb {R}},\,\, \mu _0:{\mathscr {E}}\rightarrow {\mathbb {R}},\,\,v_0:\varOmega \rightarrow {\mathbb {R}},\,\,v_1:{\mathscr {E}}\rightarrow {\mathbb {R}}$
	$\varLambda \in {\mathbb {R}}^{k\times k}$ and $w(x_b) \in {\mathbb {R}}^k$ such that (H2) and (H3) hold.
	$$\zeta (x):= \int _{x_b}^x \frac{dy}{v_0(y)}$$
*g*	$g(x,E) = v_0(x)v_1(E)$
$\mu$	$\mu (x,E) = \gamma _0(x)g(x,E) + \mu _0(E)$
*w*	$w(x) = \exp \left( \int _{x_b}^x \gamma _0(y)dy\right) \exp \left( \zeta (x)\varLambda \right) w(x_b)$
*H*	$H(E)=v_1(E)\varLambda -\mu _0(E)I$

Note that we do not lose any generality by assuming that $\varLambda$ is in Jordan normal form. Apart from the common factor $\exp \left( \int _{x_b}^x \gamma _0(y)dy\right)$ the components of *w* are therefore linear combinations of *k* building blocks of the form

5.7 $$\begin{aligned} \left( \zeta (x)\right) ^m e^{\lambda _j \zeta (x)}, \end{aligned}$$

where $j=1,2,\ldots , r,\,\, m=0,1,\ldots ,k_j-1,\,\, \sum _{j=1}^r k_j = k$. The condition (H3) guarantees that each and every building block (5.7) contributes to at least one component of *w*. By choosing $w(x_b)$ such that $d_j=1$ whenever $\theta _j$ is a generalised eigenvector of highest rank and zero otherwise, the components are (because $\varLambda$ is assumed to be in Jordan normal form) precisely these building blocks.

We now present the third and last family of the catalogue.

**Table:** 

$F_3$: generalised von Bertalanffy growth	
*k*	$k \ge 2$
Parameters	$\gamma _0:\varOmega \rightarrow {\mathbb {R}},\,\, \mu _0:{\mathscr {E}}\rightarrow {\mathbb {R}},\,\,v_0:\varOmega \rightarrow {\mathbb {R}},\,\,v_j:{\mathscr {E}}\rightarrow {\mathbb {R}},\,\, j=1,2,3.$
	$$\zeta (x):= \int _{x_b}^x \frac{dy}{v_0(y)}$$
*g*	$g(x,E) = v_0(x)\left( v_1(E)+v_2(E)\zeta (x)+v_3(E)\zeta (x)^2\right)$
$\mu$	$\mu (x,E) = \gamma _0(x)g(x,E) + \mu _0(E) +(k-1)v_3(E)\zeta (x)$
*w*	$w_j(x) = \exp \left( \int _{x_b}^x \gamma _0(y)dy\right) \zeta (x)^{j-1},\,\, j=1,2,\ldots ,k.$
*H*	$H(E)=H_0(E)-\mu _0(E)I$
	$$H_0=\left( \begin{array}{ccccc} 0 &{}-(k-1)v_3 &{}0 &{}\cdots &{}0\\ v_1 &{}v_2 &{}-(k-2)v_3 &{} &{}0 \\ 0 &{}2v_1 &{}2v_2 &{} &{}0\\ \vdots &{}0 &{}3v_1 &{} &{}0\\ &{} &{} &{} \ddots &{} \\ 0&{} \cdots &{} \cdots &{}(k-1)v_1 &{}(k-1)v_2\\ \end{array} \right) $$

It is important to realise that the parametrisation is far from unique. For instance, if we write

$$\begin{aligned} g = \frac{1}{\zeta '}\left( v_1+v_2\zeta +v_3\zeta ^2\right) \end{aligned}$$

it is not difficult to check that the linear fractional transformation

$$\begin{aligned} \eta =\frac{a \zeta +b}{c\zeta +d} \end{aligned}$$

with $ad-bc \ne 0$ yields the alternative form

$$\begin{aligned} g= \frac{1}{\eta '}\left( \widetilde{v}_1+\widetilde{v}_2\eta +\widetilde{v}_3\eta ^2\right) \end{aligned}$$

with

$$\begin{aligned} \widetilde{v}_1= & {} \frac{a^2v_1-abv_2+b^2v_3}{ad-bc},\\ \widetilde{v}_2= & {} \frac{-2acv_1+(ad+bc)v_2-2bdv_3}{ad-bc},\\ \widetilde{v}_3= & {} \frac{c^2v_1-cdv_2+d^2v_3}{ad-bc}. \end{aligned}$$

With a little bit more effort one can check that

$$\begin{aligned} \mu = \gamma _0 g+ \mu _0 +(k-1)v_3 \zeta \end{aligned}$$

becomes

$$\begin{aligned} \mu = \widetilde{\gamma }_0 g + \widetilde{\mu }_0 +(k-1) \widetilde{v}_3 \eta \end{aligned}$$

with

$$\begin{aligned} \widetilde{\gamma }_0= & {} \gamma _0 + \frac{c(k-1)\zeta '}{c\zeta +d},\\ \widetilde{\mu }_0= & {} \mu _0 -(k-1)(acv_1-bcv_2+bdv_3). \end{aligned}$$

Note that

$$\begin{aligned} \int _{x_b}^x \widetilde{\gamma }_0(y)dy = \int _{x_b}^x \gamma _0(y)dy + (k-1) \log \left( \frac{c\zeta +d}{d}\right) \end{aligned}$$

and that, accordingly,

$$\begin{aligned} \widetilde{w}_j(x)= & {} \exp \left( \int _{x_b}^x \widetilde{\gamma }_0(y)dy\right) \eta (x)^{j-1} \\= & {} \exp \left( \int _{x_b}^x \gamma _0(y)dy\right) \left( \frac{1}{d}\right) ^{k-1} (a\zeta +b)^{j-1}(c\zeta +d)^{k-j}. \end{aligned}$$

So apart from the factor $\exp \left( \int _{x_b}^x \gamma _0(y)dy\right)$ the components $\widetilde{w}_j(x)$ are linear combinations of the powers $\zeta ^\ell ,\,\, \ell =0,1,\ldots ,k-1$. We conclude that the requirement that $\{w_1,w_2,\ldots ,w_k\}$ and $\{\widetilde{w}_1,\widetilde{w}_2,\ldots ,\widetilde{w}_k\}$ are equivalent systems of output functionals is indeed satisfied.

A transformation $y=\phi (x)$ leaves the form of *g* invariant. Because of the extra factor (recall (5.4)) we have to adapt $\gamma _0$ by a factor, too. The net effect is that the integral of $\gamma _0$, and hence *w*, transforms in the standard manner.

By choosing $y=\zeta (x)$ as a new variable we eliminate the factor $v_0(x)$ and *g* becomes a quadratic polynomial in the (transformed) i-state variable. If we subsequently take $t = \log \zeta$ as the new i-state variable, we find that the growth rate of *t* has the form

$$\begin{aligned} v_1 e^{-t} + v_2 + v_3e^t. \end{aligned}$$

If, alternatively, we take $t = 2 \arctan \zeta$ as the new i-state variable, we can use the identities

$$\begin{aligned} \cos t= & {} \frac{1-\zeta ^2}{1+\zeta ^2}, \\ \sin t= & {} \frac{2 \zeta }{1+\zeta ^2}, \\ \frac{d}{d\zeta } \arctan \zeta= & {} \frac{1}{1+\zeta ^2} \end{aligned}$$

to find that the growth rate of *t* now has the form

$$\begin{aligned} v_1 (1+\cos t) +v_2 \sin t + v_3 (1-\cos t). \end{aligned}$$

We have presented the two transformations above for two reasons:

- they illustrate that appearances are deceptive (a growth rate may at first sight seem to fail to fit into the catalogue, while in fact it does),
- they play a role in the proofs.

We are now ready to state our main results.

### Theorem 5.2

If $g,\, \mu$ and *w* are of the form specified in either $F_2$ or $F_3$, then (4.33) holds for the indicated matrix *H*(*E*) and $w_1,w_2,\ldots ,w_k$ are linearly independent functions of $x \in \varOmega$.

### Theorem 5.3

Assume that $k \ge 2$ and that

(i) $g,\, \mu ,\,w$ and *H* are such that (4.33) holds,
(ii) $w_1,w_2,\ldots ,w_k$ are linearly independent functions of $x \in \varOmega$.

Then necessarily $g,\, \mu$ and *w* can be brought into the form specified in either $F_2$ or $F_3$ by a transformation of the i-state variable and a change of basis in ${\mathbb {R}}^k$.

The proofs of these two theorems are given in the next section.

## Proofs

### Some spadework

As a prelude we recall some standard theory concerning linear ODE-systems (cf. Hirsch and Smale 1974). Let $\varLambda$ be a real $k \times k$ matrix. Then $\varLambda$ defines a linear map from ${\mathbb {C}}^k$ to ${\mathbb {C}}^k$ that leaves ${\mathbb {R}}^k$, considered as the linear subspace of ${\mathbb {C}}^k$ consisting of vectors with zero imaginary part, invariant. Let $\phi _{j,1},\, j=1,2,\ldots ,r$ be a maximal set of linearly independent eigenvectors of $\varLambda$ and let $\lambda _1,\lambda _2,\ldots ,\lambda _r$ be the corresponding eigenvalues. Thus the geometric multiplicity of an eigenvalue determines how many times it is listed. We choose $\phi _{j,1} \in {\mathbb {R}}^k$ if $\lambda _j$ is real and $\phi _{j_2,1} = \overline{\phi }_{j_1,1}$ if $(\lambda _{j_1}, \lambda _{j_2})$ is a pair of non-real complex conjugate eigenvalues. Let $k_1, k_2,\ldots ,k_r$ be the corresponding multiplicities, that is, $k_j$ is the length of the Jordan block generated by $\phi _{j,1}$. For $j=1,2,\ldots ,r$ and $\ell =1,2,\ldots , k_j$, let $\phi _{j,\ell }$ be such that, with the convention $\phi _{j,0}=0$,

6.1 $$\begin{aligned} \varLambda \phi _{j,\ell } = \lambda _j \phi _{j,\ell } + \phi _{j,\ell -1} \end{aligned}$$

and, moreover, $\phi _{j,\ell } \in {\mathbb {R}}^k$ if $\lambda _j$ is real and $\phi _{j_2,\ell } = \overline{\phi }_{j_1,\ell }$ if $(\lambda _{j_1}, \lambda _{j_2})$ is a pair of non-real complex conjugate eigenvalues. Then for the complex variable *z* we have

6.2 $$\begin{aligned} e^{z\varLambda } \phi _{j,\ell } = e^{\lambda _j z} \left( \phi _{j,\ell } + \sum _{m=1}^{\ell -1} \frac{z^m}{m!} \phi _{j,\ell -m}\right) . \end{aligned}$$

The identity

6.3 $$\begin{aligned} \sum _{j=1}^r k_j = k \end{aligned}$$

holds and the set $\{\phi _{j,\ell }\}$ is a basis for ${\mathbb {C}}^k$. So for any $\psi (0) \in {\mathbb {C}}^k$ there exist coefficients $c_{j,\ell } \in {\mathbb {C}}$ such that

6.4 $$\begin{aligned} \psi (0) =\sum _{j,\ell } c_{j,\ell }\phi _{j,\ell } \end{aligned}$$

and hence

6.5 $$\begin{aligned} e^{z\varLambda } \psi (0) = \sum _{j,\ell } c_{j,\ell }\, e^{\lambda _j z} \left( \phi _{j,\ell } + \sum _{m=1}^{\ell -1} \frac{z^m}{m!} \phi _{j,\ell -m}\right) . \end{aligned}$$

When $\psi (0) \in {\mathbb {R}}^k$ we have $c_{j,\ell } \in {\mathbb {R}}$ if $\lambda _j$ is real and $c_{j_2,\ell } = \overline{c}_{j_1,\ell }$ if $(\lambda _{j_1}, \lambda _{j_2})$ is a pair of non-real complex conjugate eigenvalues. If we restrict the variable *z* to the real axis we denote it by $\xi$. We have $\exp (\xi \varLambda )\psi (0) \in {\mathbb {R}}^k$ if $\psi (0)\in {\mathbb {R}}^k$ even though the representation (6.5) possibly involves complex quantities. We want to determine conditions on $\varLambda$ and $\psi (0)$ that guarantee that the components of $\exp (\xi \varLambda )\psi (0)$, regarded as real valued functions of the real variable $\xi$ are linearly independent. To derive such conditions we first investigate the complex setting.

A preliminary yet key observation is that the functions

$$\begin{aligned} z \mapsto z^{n_1}e^{\lambda _1z} \,\, \hbox { and} \,\, z \mapsto z^{n_2}e^{\lambda _2z} \end{aligned}$$

are linearly dependent as complex functions if and only if $\lambda _1 = \lambda _2$ and $n_1= n_2$. This is obvious, because the Wronskian determinant of the two functions vanishes identically if and only if the latter condition is satisfied. By the same argument one obtains the analogous results for a finite collection of functions of the form $z \mapsto z^n \exp (\lambda z)$. Recall that for *analytic* functions the identical vanishing of the Wronskian is necessary and sufficient for linear dependence (Bôcher 1900).

#### Lemma 6.1

The components of

6.6 $$\begin{aligned} \psi (z)=e^{z\varLambda }\psi (0) \end{aligned}$$

are linearly independent functions of the complex variable *z* if and only if the following two conditions are met

(i) the eigenvalues of $\varLambda$ have geometric multiplicity one,
(ii) $c_{j,k_j} \ne 0,\,\,j=1,2,\ldots ,r$ if $\psi (0)$ is represented by (6.4).

#### Proof

As (6.5) clearly shows, each component of $\psi (z)$ is a linear combination of building blocks of the form $z^m\exp (\lambda _j z)$. According to (6.3) there are exactly *k* building blocks. To make *k* linearly independent linear combinations we need *k* linearly independent building blocks. It follows that the conditions (i) and (ii) are necessary.

By definition the components of $\psi (z)$ are linearly independent if

6.7 $$\begin{aligned} d \in {\mathbb {C}}^k, \,\,\, d\cdot \psi (z)=0 \,\,\hbox { for all}\,\, z \in {\mathbb {C}}\quad \Rightarrow \quad d=0. \end{aligned}$$

Since $\{\phi _{j,\ell }\}$ is a basis for ${\mathbb {C}}^k$, we have $d=0$ if and only if $d \cdot \phi _{j,\ell } =0$ for $j=1,2,\ldots ,r,\, \ell =1,2,\ldots ,k_j$. So we want to show that when (i) and (ii) hold, then

6.8 $$\begin{aligned} d\cdot \psi (z)=0 \,\,\hbox { for all}\,\, z \in {\mathbb {C}}\quad \Rightarrow \quad d \cdot \phi _{j,\ell } =0\,\,\hbox { for all}\,\,j,\ell . \end{aligned}$$

To do so, we deduce from (6.5) that

6.9 $$\begin{aligned} d \cdot \psi (z) = \sum _{j,\ell } c_{j,\ell }\, e^{\lambda _j z} \left( d \cdot \phi _{j,\ell } + \sum _{m=1}^{\ell -1} \frac{z^m}{m!} d \cdot \phi _{j,\ell -m}\right) . \end{aligned}$$

Because of condition (i) the right hand side of (6.9) can be identically zero only if for $j=1,2,\ldots ,r$

6.10 $$\begin{aligned} \sum _{\ell =1}^{k_j} c_{j,\ell }\, \left( d \cdot \phi _{j,\ell } + \sum _{m=1}^{\ell -1} \frac{z^m}{m!} d \cdot \phi _{j,\ell -m}\right) = 0 \quad \hbox { for all}\,\, z \in {\mathbb {C}}, \end{aligned}$$

or, equivalently,

6.11 $$\begin{aligned} \sum _{m=0}^{k_j-1}\left( \sum _{\ell =m+1}^{k_j} c_{j,\ell }\, d \cdot \phi _{j,\ell -m}\right) \frac{z^m}{m!} = 0 \quad \hbox { for all}\,\, z \in {\mathbb {C}}. \end{aligned}$$

Condition (6.11) in turn leads to

6.12 $$\begin{aligned} \sum _{\ell =m+1}^{k_j} c_{j,\ell }\, d \cdot \phi _{j,\ell -m} = 0, \quad m=0,1,\ldots ,k_j-1. \end{aligned}$$

Now assume that condition (ii) holds. Taking $m=k_j-1$ we find that necessarily $d\cdot \phi _{j,1} =0$. Next, taking $m=k_j-2$ and using $d\cdot \phi _{j,1} =0$, we find that $d\cdot \phi _{j,2} =0$. Continuing in this way we prove (6.8) and consequently (6.7) holds. This completes the sufficiency part of the proof. $\square$

#### Lemma 6.2

Let $f_j,\,\, j=1,2,\ldots ,k,$ be analytic functions defined in an open subset *A* of ${\mathbb {C}}$ that contains an interval of ${\mathbb {R}}$ and assume that $f_j(A \cap {\mathbb {R}}) \subset {\mathbb {R}}$. Then the functions $f_1,f_2,\ldots , f_k$ are linearly independent as functions of a complex variable if and only if their restrictions to $A \cap {\mathbb {R}}$ are linearly independent as functions of a real variable.

#### Proof

This follows immediately from the fact that *k* analytic functions are linearly dependent if and only if their Wronskian determinant vanishes identically (Bôcher 1900). $\square$

#### Corollary 6.3

Let $\varLambda$ be a real $k \times k$ matrix and let $\psi (0) \in {\mathbb {R}}^k$. The components of

6.13 $$\begin{aligned} \psi (\xi ) = e^{\xi \varLambda } \psi (0) \end{aligned}$$

are linearly independent functions of the real variable $\xi$ if and only if the following conditions are met

(i) the eigenvalues of $\varLambda$ have geometric multiplicity one,
(ii) if $\psi (0)$ is expressed as a linear combination of generalised eigenvectors of $\varLambda$, then the coefficients of generalized eigenvectors of highest rank are non-zero (cf. (6.4) and condition (ii) of Lemma 6.1).

Let $\varLambda$ and $\psi (0)$ satisfy the conditions (i) and (ii) of Corollary 6.3. If we order $\{\xi ^m \exp (\lambda _j \xi )\}$ (say lexicographically) as $\{h_\ell (\xi )\}_{\ell =1}^k$, then there exist coefficients $d_{j\ell }$ such that

6.14 $$\begin{aligned} \psi _j(\xi ) = \sum _{\ell =1}^k d_{j\ell } h_\ell (\xi ). \end{aligned}$$

Let $$c \in {\mathbb {R}}^k$$. Then

6.15 $$\begin{aligned} c \cdot \psi (\xi ) = \sum _{j=1}^k c_{j} \psi _j(\xi ) = \sum _{\ell =1}^k\left( \sum _{j=1}^k c_j d_{j\ell }\right) h_\ell (\xi ). \end{aligned}$$

By the linear independence of $\{h_\ell \}_{\ell =1}^k$ we have that $c \cdot \psi =0$ if and only if $\sum _{j=1}^k c_j d_{j\ell } =0$ for $\ell =1,2,\ldots ,k$. By Corollary 6.3$c \cdot \psi =0$ implies $c=0$. This translates into

6.16 $$\begin{aligned} cD = 0 \quad \Rightarrow \quad c=0, \end{aligned}$$

where *D* is the $k \times k$ matrix with elements $d_{j\ell }$. We conclude that *D* is invertible. So when conditions (i) and (ii) of Corollary 6.3 hold, there exists an invertible transformation *D* that relates the vector $\psi (\xi )= \exp (\xi \varLambda )\psi (0)$ to the vector $h(\xi )$ with components $h_\ell (\xi )$.

Note that $h_{\ell _1}$ is complex valued if $\mathrm{Im}\, \lambda _{j_1} \ne 0$, but that in that case there exists $\ell _2$ such that $h_{\ell _2}(\xi ) =\overline{h}_{\ell _1}(\xi )$ and, moreover, $d_{j\ell _2} = \overline{d}_{j\ell _2}$.

### Proof of Theorem 5.2

#### The model family $F_2$

The proof that (4.33) holds for $F_2$ amounts to a straightforward verification. The linear independence of $\{w_1,w_2,\ldots ,w_k\}$ is guaranteed by Corollary 6.3. $\square$

#### The model family $F_3$

Let $g,\, \mu , w, \zeta$ be as specified in $F_3$. Since

$$\begin{aligned} \zeta (x) = \int _{x_b}^x \frac{dy}{v_0(y)}, \end{aligned}$$

$\zeta (x)$ is not constant and as a consequence the components

6.17 $$\begin{aligned} w_j(x) = \exp \left( \int _{x_b}^x \gamma _0(y)dy\right) \zeta (x)^{j-1} \end{aligned}$$

of *w* are linearly independent functions of *x*.

To verify (4.33), which under $F_3$ takes the form

6.18 $$\begin{aligned} g(x,E)\frac{dw}{dx}(x) = H_0(E)w(x) + (\mu (x,E)-\mu _0(E))w(x), \end{aligned}$$

note that

6.19 $$\begin{aligned} \frac{d}{dx} \left( \zeta (x)\right) ^{j-1} = (j-1)\left( \zeta (x)\right) ^{j-2} \frac{1}{v_0(x)} \end{aligned}$$

and hence

6.20 $$\begin{aligned} g(x,E)\frac{d}{dx} \left( \zeta (x)\right) ^{j-1}= & {} (j-1)\left( v_1(E)\left( \zeta (x)\right) ^{j-2} + v_2(E)\left( \zeta (x)\right) ^{j-1} \right. \nonumber \\&\left. +v_3(E)\left( \zeta (x)\right) ^{j} \right) . \end{aligned}$$

Differentiating (6.17) one obtains

6.21 $$\begin{aligned} \frac{d}{dx}w_j(x)= \gamma _0(x)w_j(x) + e^{\int _{x_b}^x \gamma _0(y)dy}\frac{d}{dx} \left( \zeta (x)\right) ^{j-1} \end{aligned}$$

and multiplying (6.21) by *g*(*x*, *E*), taking (6.17), (6.19) and the form of $\mu (x,E)$ specified in $F_3$ into account, one obtains

6.22 $$\begin{aligned} g(x,E)\frac{d}{dx}w_j(x)= & {} \gamma _0(x)g(x,E) w_j(x) \nonumber \\&+e^{\int _{x_b}^x \gamma _0(y)dy} (j-1)\left( v_1(E)\zeta (x)^{j-2}\right. \nonumber \\&\left. +v_2(E)\zeta (x)^{j-1} +v_3(E)\zeta (x)^j\right) \nonumber \\= & {} (\mu (x,E)-\mu _0(E))w_j(x) \nonumber \\&+e^{\int _{x_b}^x \gamma _0(y)dy}\left( (j-1)v_1(E)\zeta (x)^{j-2} +(j-1)v_2(E)\zeta (x)^{j-1}\right. \nonumber \\&\left. -(k-j)v_3(E)\zeta (x)^j\right) \end{aligned}$$

The *j*th component of the right hand side of (6.18) and the right hand side of (6.22) contain the common term $(\mu (x,E)-\mu _0(E))w_j(x)$. Thus to verify that (6.18) holds, we only have to check that the *j*th component of the vector obtained by applying the matrix $H_0(E)$ as specified in $F_3$ to the vector $\left( 1\,\, \zeta (x)\,\, \zeta (x)^2\,\,\ldots \,\, \zeta (x)^{k-1}\right) ^T$ is

$$\begin{aligned} (j-1)v_1(E)\zeta (x)^{j-2} +(j-1)v_2(E)\zeta (x)^{j-1}-(k-j)v_3(E)\zeta (x)^j \end{aligned}$$

and this is obviously the case. $\square$

### Proof of Theorem 5.3

For $k=2$ and for given *H* and *w* we may consider (4.33) as two linear equations in the two unknowns *g* and $\mu$. The solution has the form given in $F_3$ with $\zeta , \, v_i,\, \gamma _0$ and $\mu _0$ expressed in terms of *w* and *H*. This computation provides a strong indication that Theorem 5.3 is correct, but it does not yield a proof.

Our strategy is to focus first on the fixed value $E_0$ of *E* such that $g(x,E_0) >0$ for all $x \in \varOmega$. The existence of such an $E_0$ is guaranteed by Assumption 5.1. This enables us to show that *w* is a transformed version of a matrix exponential and hence consists of building blocks of the form $\xi ^m \exp (\lambda \xi )$. After that we consider general *E* and investigate the consequences for (4.33).

Define

6.23 $$\begin{aligned} V(x) := \exp \left( -\int _{x_b}^x \frac{\mu (y,E_0)}{g(y,E_0)}dy \right) w(x). \end{aligned}$$

Then (4.33) is equivalent to

6.24 $$\begin{aligned} g(x,E)V'(x) =\left( H(E)+\widetilde{\mu }(x,E)\right) V(x) \end{aligned}$$

with

6.25 $$\begin{aligned} \widetilde{\mu }(x,E):= \mu (x,E) -\frac{g(x,E)}{g(x,E_0)}\mu (x,E_0) \end{aligned}$$

and hence

6.26 $$\begin{aligned} \widetilde{\mu }(x,E_0)= 0 \quad \hbox { for all}\,\, x \in \varOmega . \end{aligned}$$

Next define

6.27 $$\begin{aligned} \tau (x) := \int _{x_b}^x \frac{dy}{g(y,E_0)} \end{aligned}$$

and

6.28 $$\begin{aligned} \varPhi (t):= V\left( \tau ^{-1}(t)\right) . \end{aligned}$$

Then (6.24) is equivalent to

6.29 $$\begin{aligned} \widetilde{g}(t,E) \varPhi '(t) = \left( H(E)+\widetilde{\mu }\left( \tau ^{-1}(t),E\right) \right) \varPhi (t) \end{aligned}$$

with

6.30 $$\begin{aligned} \widetilde{g}(t,E) =\frac{g\left( \tau ^{-1}(t),E\right) }{g\left( \tau ^{-1}(t),E_0\right) } \end{aligned}$$

and hence

6.31 $$\begin{aligned} \widetilde{g}(t,E_0)=1 \quad \hbox { for all}\,\, t \in \tau (\varOmega ). \end{aligned}$$

It follows that

6.32 $$\begin{aligned} \varPhi (t) = e^{tH(E_0)}\varPhi (0) \end{aligned}$$

and hence necessarily $H(E_0)$ and $\varPhi (0)$ satisfy the conditions (i) and (ii) of Corollary 6.3 (as otherwise *w* would not consist of *k* linearly independent functions of *x*).

Let $\lambda _1,\lambda _2,\ldots , \lambda _r$ denote the eigenvalues of $H(E_0)$ with multiplicities $k_1,k_2,\ldots ,k_r$ such that $\sum _{p=1}^r k_p =k$. If we order $\{t^m\exp (\lambda _p t)\}$ lexicographically as $\{h_\ell (t)\}_{\ell =1}^k$, the analogue of (6.14) reads

6.33 $$\begin{aligned} \varPhi (t) = DX(t). \end{aligned}$$

Applying the matrix $D^{-1}$ to (6.29) we find

6.34 $$\begin{aligned} \widetilde{g}(t,E)X'(t) -\widetilde{\mu }\left( \tau ^{-1}(t),E \right) X(t) = D^{-1}H(E)DX(t). \end{aligned}$$

Every component of the right hand side of (6.34) is a linear combination, with *E*-dependent coefficients, of the building blocks

6.35 $$\begin{aligned} h_\ell (t) = t^m e^{\lambda _p t}. \end{aligned}$$

The $\ell$th component of the left hand side of (6.34) reads

$$\begin{aligned} \widetilde{g}(t,E)\left( m t^{m-1} + \lambda _p t^m \right) e^{\lambda _p t} - \widetilde{\mu }\left( \tau ^{-1}(t),E \right) t^m e^{\lambda _p t}. \end{aligned}$$

Hence (6.34) implies that for $p=1,2,\ldots ,r,\, m=0,1,\ldots ,k_p-1$ one has

6.36 $$\begin{aligned} \left( m t^{m-1} + \lambda _p t^m \right) \widetilde{g}(t,E) - t^m \widetilde{\mu }\left( \tau ^{-1}(t),E \right) = \sum _{j=1}^r\sum _{\ell =0}^{k_j-1} c_{j\ell } t^\ell e^{(\lambda _j-\lambda _p)t},\nonumber \\ \end{aligned}$$

where the coefficients $c_{j\ell }$ depend on $E,\,m$ and *p*.

Apart from $\widetilde{g},\, \widetilde{\mu }$ and powers of *t*, the identity (6.36) involves exponential functions with exponents from the set

6.37 $$\begin{aligned} U_p:=\left\{ \lambda _j - \lambda _p:j=1,2,\ldots ,r\right\} . \end{aligned}$$

Since (6.36) should hold for $p=1,2,\ldots ,r$, we are particularly interested in a characterisation of

$$\begin{aligned} \bigcap _{p=1}^r U_p. \end{aligned}$$

As already observed above, we know that necessarily

6.38 $$\begin{aligned} \lambda _j \ne \lambda _\ell \quad \hbox { if} \quad j \ne \ell . \end{aligned}$$

#### Lemma 6.4

Let $$r \ge 2$$. Then

$$\begin{aligned} \bigcap _{p=1}^r U_p = \left\{ 0\right\} . \end{aligned}$$

#### Proof

Let $\alpha \in \bigcap _{p=1}^r U_p$ and assume that $\alpha \ne 0$. Since $\alpha \in U_j$ there exists an $m=m(j)$ such that

6.39 $$\begin{aligned} \lambda _m = \lambda _j + \alpha . \end{aligned}$$

Because of (6.38), there is at most one *m* for which (6.39) holds. We write $m=s(j)$, with *s* standing for “successor”, and thus define a map $s:\{1,2,\ldots ,r\} \rightarrow \{1,2,\ldots ,r\}$. The sequence $1, s(1), s^2(1),\ldots$ takes values in a finite set so necessarily some value is taken at least twice. This implies that

6.40 $$\begin{aligned} s^\ell (j) = j \end{aligned}$$

for some *j* and $\ell \ge 1$. But (6.40) means that

6.41 $$\begin{aligned} \lambda _j=\lambda _j+ \ell \alpha \end{aligned}$$

so after all, $\alpha = 0$. $\square$

Suppose that, with appropriate numbering of the eigenvalues of $H(E_0)$, we have

6.42 $$\begin{aligned} \lambda _j = \lambda _1 +(j-1)\alpha . \end{aligned}$$

Then

6.43 $$\begin{aligned} U_p = \left\{ (j-p)\alpha :j=1,2,\ldots ,r\right\} \end{aligned}$$

and hence

6.44 $$\begin{aligned} \bigcap _{p=2}^r U_p = \{-\alpha , 0\}. \end{aligned}$$

We now show that the existence of a non-zero element $-\alpha$ in $\bigcap _{p=2}^r U_p$ implies that (6.42) holds, or, in other words, that (6.42) and (6.44) are equivalent.

#### Lemma 6.5

Let $r \ge 2$. Either $\bigcap _{p=2}^r U_p = \{0\}$ or, possibly after renumbering the $\lambda$’s, (6.42) holds.

#### Proof

Let $-\alpha \in \bigcap _{p=2}^r U_p$ and assume that $\alpha \ne 0$. As in the proof of Lemma 6.4 we define the successor map *s*. But now *s* is defined on $\{2,3,\ldots ,r\}$ while taking values in $\{1,2,\ldots ,r\}$. If the sequence $j, s(j), s^2(j),\ldots$ takes values only in $\{2,3,\ldots ,r\}$, it necessarily becomes periodic and again we conclude that actually $\alpha = 0$. Hence $\alpha \ne 0$ requires that the sequence hits 1 after finitely many steps, implying that

$$\begin{aligned} \lambda _1 = \lambda _j - m(j)\alpha \end{aligned}$$

which with appropriate numbering of the $\lambda$’s amounts to (6.42). $\square$

In our analysis of (6.36) we shall also subtract the identity with $p=p_1$ from the identity with $p=p_2$. This motivates us to consider the intersection of all unions $U_{p_1}\bigcup U_{p_2}$ with $p_1 \ne p_2$.

#### Lemma 6.6

If

6.45 $$\begin{aligned} \alpha \in \bigcap _{p_1 \ne p_2} \left( U_{p_1}\bigcup U_{p_2} \right) {\setminus } \{0\} \end{aligned}$$

then, with appropriate numbering of the $\lambda$’s, (6.42) holds and

6.46 $$\begin{aligned} \bigcap _{p_1 \ne p_2} \left( U_{p_1}\bigcup U_{p_2} \right) = \{-\alpha ,0,\alpha \}. \end{aligned}$$

#### Proof

Assume (6.45). We claim that there can be at most one index *p* such that $\alpha \notin U_p$, that is,

6.47 $$\begin{aligned} \alpha \ne \lambda _j - \lambda _p \quad \hbox { for} \quad j=1,2,\ldots ,r. \end{aligned}$$

Indeed, if (6.47) would hold for $p=q_1$ as well as for $p=q_2$, with $q_1 \ne q_2$, then

$$\begin{aligned} \alpha \notin U_{q_1}\bigcup U_{q_2} \end{aligned}$$

and hence certainly

$$\begin{aligned} \alpha \notin \bigcap _{p_1 \ne p_2} \left( U_{p_1}\bigcup U_{p_2} \right) . \end{aligned}$$

Lemma 6.4 implies that at least one index *p* must exist such that $\alpha \notin U_p$. So there exists a unique index *p* such that $\alpha \notin U_p$. Let us call it the exceptional index.

Renumber the $\lambda$’s such that the exceptional index is 1. Then $\alpha \in \bigcap _{p=2}^r U_p$ and we can apply Lemma 6.5 to deduce that (6.42) holds. It follows that elements of

$$\begin{aligned} \bigcap _{p_1 \ne p_2} \left( U_{p_1}\bigcup U_{p_2} \right) \end{aligned}$$

are of the form $m\alpha$ with $1-r \le m \le r-1$. The number 0 belongs to $U_p$ for all *p*, the number $\alpha$ for all $p\ne r$, and the number $-\alpha$ for all $p\ne 1$. So the right hand side of (6.46) is a subset of the left hand side. If $m \ge 2$, the number $m\alpha$ does not belong to $U_p$ for $p=r-1, r$ and if $m \le -2$ it does not belong to $U_p$ for $p=1,2$. We conclude that (6.46) holds. $\square$

#### Lemma 6.7

Let $r \ge 2$ and assume that

6.48 $$\begin{aligned} \bigcap _{p_1 \ne p_2} \left( U_{p_1}\bigcup U_{p_2} \right) = \{0\}. \end{aligned}$$

Then both $t \mapsto \widetilde{g}(t,E)$ and $t \mapsto \widetilde{\mu }\left( \tau ^{-1}(t),E\right)$ are constant functions.

#### Proof

Consider (6.36) with $m=0$ for $p=p_1$ and for $p=p_2$. By subtraction we find

6.49 $$\begin{aligned} \left( \lambda _{p_2}-\lambda _{p_1}\right) \widetilde{g}(t,E) = \sum _{j=1}^r \sum _{\ell =0}^{k_j-1} \left( c_{j\ell }(p_2)e^{\left( \lambda _j-\lambda _{p_2}\right) t}-c_{j\ell }(p_1)e^{\left( \lambda _j-\lambda _{p_1}\right) t}\right) t^\ell .\nonumber \\ \end{aligned}$$

The identity (6.49) holds for all $p_1$ and $p_2$ with $p_1\ne p_2$ (and of course trivially for $p_1=p_2$). It therefore follows from (6.48) that all exponentials with non-zero exponent in (6.49) must have coefficient zero, that is, necessarily

$$\begin{aligned} c_{j\ell }(p_m) =0 \quad \hbox { if} \quad j \ne p_m. \end{aligned}$$

It follows that the map $t \mapsto \widetilde{g}(t,E)$ is a polynomial of degree $d_1 \le \max \{k_p\} -1$.

Returning to (6.36) with $m=0$, we express $\widetilde{\mu }$ in terms of $\lambda _p \widetilde{g}$ and the sum at the right hand side. From the arbitrariness of *p* and Lemma 6.4 we deduce that the map $t \mapsto \widetilde{\mu }\left( \tau ^{-1}(t),E\right)$, too, is a polynomial and that its degree $d_2$ does not exceed $\max \{k_p\}-1$. Hence the map

$$\begin{aligned} t \mapsto \left( \lambda _p \widetilde{g}(t,E)-\widetilde{\mu }\left( \tau ^{-1}(t),E\right) \right) t^m+mt^{m-1} \widetilde{g}(t,E) \end{aligned}$$

is a polynomial of degree $d_3 =\max \{d_1,d_2\}+m$ (unless $\lambda _p \widetilde{g}-\widetilde{\mu } =0$, but this can be the case for at most one value of *p*).

The right hand side of (6.36) is a polynomial in *t* if and only if $c_{j\ell }(p)=0$ for $j \ne p$ and, if it is, the degree is at most $k_p-1$. We conclude that

$$\begin{aligned} \max \{d_1,d_2\} + m \le k_p -1 \end{aligned}$$

and consequently, by taking $m=k_p-1$, that

$$\begin{aligned} \max \{d_1,d_2\} =0. \end{aligned}$$

$$\square $$

#### Corollary 6.8

Let $r\ge 2$ and assume that (6.48) holds. Then $g,\, \mu ,\, w$ and *H* are of the form specified in $F_2$ with

$$\begin{aligned} v_0(x)= & {} g(x,E_0), \\ v_1(E)= & {} \widetilde{g}(\tau (x),E), \\ \gamma _0(x)= & {} \frac{\mu (x,E_0)}{g(x,E_0)}, \\ \mu _0(E)= & {} \widetilde{\mu }(x,E), \\ \zeta (x)= & {} \tau (x) = \int _{x_b}^x \frac{dy}{g(y,E_0)}, \\ \varLambda= & {} H(E_0). \end{aligned}$$

#### Proof

From (6.30) we know that

6.50 $$\begin{aligned} g(x,E) = g(x,E_0)\widetilde{g}(\tau (x),E) \end{aligned}$$

and by Lemma 6.7 the second factor on the right hand side of (6.50) is constant as a function of its first argument, so with the given definitions of $v_0$ and $v_1$, the function *g* is indeed of the form specified in $F_2$.

Likewise it follows from (6.25) that

6.51 $$\begin{aligned} \mu (x,E) = \frac{\mu (x,E_0)}{g(x,E_0)}g(x,E) + \widetilde{\mu }(x,E). \end{aligned}$$

The last term on the right hand side of (6.51) is, by Lemma 6.7, constant as a function of its first argument and hence $\mu$, too, is of the form specified in $F_2$ with $\gamma _0$ and $\mu _0$ as listed.

From (6.23), (6.28) and (6.32) we deduce that

6.52 $$\begin{aligned} w(x) = e^{\int _{x_b}^x \gamma _0(y)dy}\varPhi ((\tau (x)) = e^{\int _{x_b}^x \gamma _0(y)dy} e^{\tau (x)H(E_0)} \varPhi (0), \end{aligned}$$

where $\tau (x)$ is defined by (6.27). Now recall (6.31) and translate it into $v_1(E_0)=1$. It follows that

6.53 $$\begin{aligned} \tau (x) = \int _{x_b}^x \frac{dy}{g(y,E_0)} = \int _{x_b}^x \frac{dy}{v_0(y)} \end{aligned}$$

Substituting (6.53) into (6.52) and taking into account that $\varLambda =H(E_0)$ because $v_1(E_0)=1$ and $\mu _0(E_0)=0$ by (6.26), we see that *w* is of the form specified in $F_2$. $\square$

#### Lemma 6.9

Let $r=1,\, k\ge 2$. Then necessarily

6.54 $$\begin{aligned} \widetilde{g}(t,E)= & {} v_1(E) + v_2(E)t+v_3(E)t^2, \end{aligned}$$

6.55 $$\begin{aligned} \widetilde{\mu }\left( \tau ^{-1}(t),E\right)= & {} \lambda \widetilde{g}(t,E) + \mu _0(E) + (k-1)v_3(E)t. \end{aligned}$$

#### Proof

Because we assume that $r=1$, we can write (6.36) as

6.56 $$\begin{aligned} \left( \lambda \widetilde{g}(t,E) - \widetilde{\mu }\left( \tau ^{-1}(t),E\right) \right) t^m + mt^{m-1} \widetilde{g}(t,E) = \sum _{\ell =0}^{k-1} c_\ell t^\ell , \end{aligned}$$

with $c_\ell$ depending on *E* and *m*. We make the dependence on *m* explicit by writing $c_\ell (m)$.

If we take $m=0$ and multiply (6.56) by *t* we obtain

6.57 $$\begin{aligned} \left( \lambda \widetilde{g}(t,E) - \widetilde{\mu }\left( \tau ^{-1}(t),E\right) \right) t = \sum _{\ell =0}^{k-1} c_\ell (0) t^{\ell +1} \end{aligned}$$

while for $m=1$, (6.56) takes the form

6.58 $$\begin{aligned} \left( \lambda \widetilde{g}(t,E) - \widetilde{\mu }\left( \tau ^{-1}(t),E\right) \right) t + \widetilde{g}(t,E) = \sum _{\ell =0}^{k-1} c_\ell (1) t^\ell . \end{aligned}$$

Subtracting (6.57) from (6.58) we find that $\widetilde{g}(t,E)$ is a polynomial in *t*.

Next we observe that (6.56) with $m=k-2$ and multiplied by *t* becomes

6.59 $$\begin{aligned} \left( \lambda \widetilde{g}(t,E) - \widetilde{\mu }\left( \tau ^{-1}(t),E\right) \right) t^{k-1} +(k-2)t^{k-2}\widetilde{g}(t,E) = \sum _{\ell =0}^{k-1} c_\ell t^{\ell +1} \end{aligned}$$

and that for $m=k-1$ the identity (6.56) reads

6.60 $$\begin{aligned} \left( \lambda \widetilde{g}(t,E) - \widetilde{\mu }\left( \tau ^{-1}(t),E\right) \right) t^{k-1} +(k-1)t^{k-2}\widetilde{g}(t,E) = \sum _{\ell =0}^{k-1} c_\ell t^\ell . \end{aligned}$$

If we subtract (6.60) from (6.59), we find that $t^{k-2} \widetilde{g}(t,E)$ is a polynomial in *t* of degree at most *k*. Since $\widetilde{g}(t,E)$ itself is a polynomial in *t*, we conclude that its degree is at most 2, that is, (6.54) must hold.

It follows from (6.54) and (6.60) that

$$\begin{aligned} \left( \lambda \widetilde{g}(t,E) - \widetilde{\mu }\left( \tau ^{-1}(t),E\right) \right) t^{k-1} \end{aligned}$$

is a polynomial of degree at most *k* and hence

6.61 $$\begin{aligned} \lambda \widetilde{g}(t,E) - \widetilde{\mu }\left( \tau ^{-1}(t),E\right) =-\mu _0(E) -\mu _1(E)t. \end{aligned}$$

Substituting (6.61) into (6.60) shows that necessarily

$$\begin{aligned} \mu _1(E) = (k-1)v_3(E). \end{aligned}$$

This yields (6.55). $$\square $$

We say that a square matrix is in *subdiagonal Jordan form* if the entries on the main diagonal equal $\lambda$ and the entries on the subdiagonal (that is, the entries immediately under the diagonal) equal 1 and all other entries equal 0.

#### Corollary 6.10

Let $r=1,\, k\ge 2$. Assume that $H(E_0)$ is in subdiagonal Jordan form and let $\varPhi (0) = (1\, 0\, \cdots \, 0)^T$. Then $g,\,\mu ,\, w$ and *H* are as specified in $F_3$.

#### Proof

Because $H(E_0)$ is in subdiagonal Jordan form and $\varPhi (0) = (1\, 0\, \cdots \, 0)^T$, we have

6.62 $$\begin{aligned} \varPhi (t) = e^{\lambda t} \left( \begin{array}{c} 1 \\ t \\ \vdots \\ t^{k-1} \end{array} \right) . \end{aligned}$$

Combining (6.30) and (6.54) we find that *g* has the form specified in $F_3$ with

$$\begin{aligned} v_0(x) =g(x,E_0) \end{aligned}$$

and hence

$$\begin{aligned} \zeta (x) =\tau (x), \end{aligned}$$

where $\tau (x)$ is defined by (6.27). Combining (6.25) and (6.55) we find that $\mu$ is of the form specified in $F_3$ with

$$\begin{aligned} \gamma _0(x) = \frac{\mu (x,E_0)+\lambda }{g(x,E_0)}. \end{aligned}$$

Combining (6.23), (6.28) and (6.62) and taking (6.27) into account, we see that *w* is of the form specified in $F_3$.

Finally we deduce the form of *H*(*E*) by substituting what we know about $g,\,\mu$ and *w* into (4.33). $\square$

#### Lemma 6.11

Let $r \ge 2$ and assume that

$$\begin{aligned} \alpha \in \bigcap _{p_1 \ne p_2} \left( U_{p_1}\bigcup U_{p_2} \right) {\setminus } \{0\}. \end{aligned}$$

Then necessarily $k_j=1$ for $j=1,2,\ldots ,r$ and hence $r=k$ and, moreover, there exist $u_j(E), \, j=1,2,3,4$ such that

6.63 $$\begin{aligned} \widetilde{g}(t,E)= & {} u_1(E) e^{-\alpha t} + u_2(E) + u_3(E) e^{\alpha t}, \end{aligned}$$

6.64 $$\begin{aligned} \widetilde{\mu }\left( \tau ^{-1}(t),E\right)= & {} \lambda _1 u_1(E) e^{-\alpha t} +u_4(E)+\lambda _k u_3(E) e^{\alpha t}, \end{aligned}$$

where the $\lambda$’s are numbered such that (6.42) holds. One has

6.65 $$\begin{aligned} u_1(E_0) = 0, \quad u_2(E_0) = 1, \quad u_3(E_0) = 0, \quad u_4(E_0) = 0. \end{aligned}$$

#### Proof

Recall that in Lemma 6.6 we established that, with appropriate numbering, (6.42) holds and that

$$\begin{aligned} \bigcap _{p_1 \ne p_2} \left( U_{p_1}\bigcup U_{p_2} \right) = \{-\alpha ,0,\alpha \}. \end{aligned}$$

From (6.49) we now deduce that

$$\begin{aligned} \widetilde{g}(t,E) = P_-(t,E) e^{-\alpha t} + P_0(t,E) + P_+(t,E)e^{\alpha t}, \end{aligned}$$

where $P_-,\,P_0$ and $P_+$ are all polynomials in *t* of degree at most $\max \{k_p\}-1$. Next (6.36) with $m=0$ and Lemma 6.4 imply that

$$\begin{aligned} \widetilde{\mu }\left( \tau ^{-1}(t),E\right) = Q_-(t,E) e^{-\alpha t} + Q_0(t,E) + Q_+(t,E)e^{\alpha t}, \end{aligned}$$

where, likewise, $Q_-,\,Q_0$ and $Q_+$ are polynomials in *t* of degree at most $\max \{k_p\}-1$.

We rewrite (6.36) with $p=1$ and $m \le k_1-1$ as

6.66 $$\begin{aligned}&t^m \left( (\lambda _1P_-(t,E)-Q_-(t,E))e^{-\alpha t} +\lambda _1P_0(t,E)-Q_0(t,E)\right. \nonumber \\&\qquad \left. + (\lambda _1P_+(t,E)-Q_+(t,E))e^{\alpha t}\right) \nonumber \\&\qquad + mt^{m-1} \left( P_-(t,E)e^{-\alpha t} + P_0(t,E) + P_+(t,E)e^{\alpha t}\right) \nonumber \\&\quad = \sum _{j=1}^r \sum _{\ell =0}^{k_j-1} c_{j\ell }t^\ell e^{(j-1)\alpha t}. \end{aligned}$$

At the right hand side we have terms with factors $e^{\alpha t}, e^{2\alpha t}, \ldots$, but not with $e^{-\alpha t}$. So necessarily

(i) $$\lambda _1 P_-(t,E) - Q_-(t,E) =0$$,
(ii) if $k_1 >1$, then $P_-(t,E) =0$.

By considering the analogue of (6.66) for $p=r$, we similarly find

(iii) $$\lambda _r P_+(t,E) - Q_+(t,E) =0$$,
(iv) if $k_r >1$, then $P_+(t,E) =0$.

Next we consider the analogue of (6.66) for $p=2$ and concentrate on the terms with factor $e^{-\alpha t}$. At the right hand side $e^{-\alpha t}$ is multiplied by a polynomial of degree $k_1-1$ while at the left hand side *m* might go up to $k_2-1$ and $\lambda _2P_-(t,E)-Q_-(t,E)\not \equiv 0$ unless both $P_-$ and $Q_-$ are identically equal to zero. So if $P_-$ and $Q_-$ are nontrivial, $k_1=1$ implies $k_2=1$. Likewise we may go down from *r* to $r-1$ and concentrate on terms with factor $e^{\alpha t}$. We find that if $P_+$ and $Q_+$ are nontrivial, $k_r=1$ implies $k_{r-1}=1$. Continuing in this way we conclude that, unless $P_-,\, Q_-,\,P_+$ and $Q_+$ are all identically equal to zero, necessarily all $k_i$’s are equal to 1. It follows that $P_-,\, Q_-,\,P_0,\, Q_0,\,P_+$ and $Q_+$ all have degree zero, that is, they are constant functions of *t*.

Finally (6.65) follows from (6.26) and (6.31). $\square$

So far it has been tacitly understood that $g,\, \mu$ and *w* are real valued functions and that the matrix $H(E_0)$ has real entries. But the fact that $H(E_0)$ is a real matrix does not preclude complex eigenvalues, it only implies that non-real eigenvalues occur in complex conjugate pairs. If we allow $\alpha$ to be complex, the relation (6.42) tells us that the eigenvalues lie on a straight line in the complex plane. This is compatible with the requirement of eigenvalues occurring in complex conjugate pairs if and only if either $\alpha$ is real or $\alpha = i\beta$ for some real $\beta$. We conclude that either $\alpha$ is real and all eigenvalues are real or $\alpha = i\beta$ and the eigenvalues lie on a line parallel to the imaginary axis.

#### Corollary 6.12

Let $$r \ge 2$$. If

$$\begin{aligned} \bigcap _{p_1 \ne p_2} \left( U_{p_1}\bigcup U_{p_2} \right) \end{aligned}$$

contains an $\alpha \in {\mathbb {R}}{\setminus }\{0\}$, then $g,\, \mu ,\, w$ and *H* have the form specified in $F_3$.

#### Proof

Combining (6.30) with Lemma 6.11 we find that

$$\begin{aligned} g(x,E)= & {} g(x,E_0)\left( u_1(E)e^{-\alpha \tau (x)} +u_2(E)+u_3(E)e^{\alpha \tau (x)} \right) \\= & {} \frac{1}{\alpha }g(x,E_0)e^{-\alpha \tau (x)} \left( \alpha u_1(E) +\alpha u_2(E)e^{\alpha \tau (x)} + \alpha u_3(E)\left( e^{\alpha \tau (x)}\right) ^2\right) \end{aligned}$$

and thus *g* has the form specified in $F_3$ with

6.67 $$\begin{aligned} v_0(x)= & {} \frac{1}{\alpha }g(x,E_0)e^{-\alpha \tau (x)}, \nonumber \\ v_i(E)= & {} \alpha u_i(E), \quad i=1,2,3, \nonumber \\ \zeta (x)= & {} e^{\alpha \tau (x)}. \end{aligned}$$

Note that by (6.27)

$$\begin{aligned} \zeta '(x) = \alpha \frac{1}{g(x,E_0)}e^{\alpha \tau (x)} = \frac{1}{v_0(x)}, \end{aligned}$$

so

$$\begin{aligned} \zeta (x) = \int _{x_b}^x \frac{dy}{v_0(y)} \end{aligned}$$

as it should.

From (6.25) and (6.64) it follows that

$$\begin{aligned} \mu (x,E)= & {} \frac{\mu (x,E_0)}{g(x,E_0)}g(x,E)+\lambda _1\left( u_1(E)e^{-\alpha \tau (x)}+u_3(E)e^{\alpha \tau (x)} \right) \\&+u_4(E)+(k-1)\alpha u_3(E)e^{\alpha \tau (x)} \\= & {} \gamma _0(x)g(x,E) +\mu _0(E) +(k-1)v_3(E)\zeta (x), \end{aligned}$$

where, in addition to (6.67), we have defined

$$\begin{aligned} \gamma _0(x)= & {} \frac{\mu (x,E_0) +\lambda _1}{g(x,E_0)}, \\ \mu _0(E)= & {} u_4(E) - \lambda _1 u_2(E). \end{aligned}$$

Now recall (6.32) and (6.42). Since all eigenvalues of $H(E_0)$ have multiplicity one, we can choose for $H(E_0)$ the diagonal matrix with the eigenvalues on the diagonal. With the choice $\varPhi _j(0)=1,\, j=1,2,\ldots , k$, we have

$$\begin{aligned} \varPhi _j(t) = e^{\lambda _1 t} e^{(j-1)\alpha t}. \end{aligned}$$

A combination of (6.23) and (6.28) next yields

6.68 $$\begin{aligned} w(x) = e^{\int _{x_b}^x\frac{\mu (y,E_0)}{g(y,E_0)}dy} \varPhi (\tau (x)) \end{aligned}$$

and the definitions of $\zeta ,\, \gamma _0$ and $\varPhi _j$ guarantee that (6.68) is exactly of the form specified in $F_3$.

Plugging in the information we now have about $g,\, \mu$ and *w* into (4.33), we find that *H*(*E*) has the form specified in $F_3$. Note that (6.65) of Lemma 6.11 implies that $v_1(E_0) = \alpha u_1(E_0)=0,\, v_2(E_0)= \alpha u_2(E_0)=\alpha$ and $v_3(E_0) = \alpha u_3(E_0)=0$ which shows that $H(E_0)$ is indeed the diagonal matrix specified above. $\square$

When

$$\begin{aligned} \bigcap _{p_1 \ne p_2} \left( U_{p_1}\bigcup U_{p_2} \right) \end{aligned}$$

contains a purely imaginary number $\alpha =i\beta$ ($\beta$ real and $\beta \ne 0$), we have $\lambda _k = \overline{\lambda }_1$ and hence $\mathrm{Im}\, \lambda _1 = -\frac{k-1}{2}\beta$. It follows that

6.69 $$\begin{aligned} \lambda _j = \mathrm{Re}\,\lambda _1 +\left( j - \frac{k+1}{2}\right) i\beta . \end{aligned}$$

In (6.63) we now allow $u_1(E)$ to be complex while requiring $u_3(E) = \overline{u_1(E)}$. Using (6.30) we find

6.70 $$\begin{aligned} g(x,E)= & {} g(x,E_0)\left( u_1(E)e^{-i\beta \tau (x)} + u_2(E)+\overline{u_1(E)}e^{i\beta \tau (x)}\right) \nonumber \\= & {} g(x,E_0)\left( c_1(E)\cos \beta \tau (x) + c_2(E)\sin \beta \tau (x)+u_2(E)\right) , \end{aligned}$$

where

6.71 $$\begin{aligned} c_1(E):= & {} 2\, \mathrm{Re}\,u_1(E),\nonumber \\ c_2(E):= & {} 2\, \mathrm{Im}\,u_1(E). \end{aligned}$$

The well-known trigonometric identities

$$\begin{aligned} \cos \theta= & {} \cos ^2\frac{1}{2}\theta - \sin ^2\frac{1}{2}\theta \\ \sin \theta= & {} 2\sin \frac{1}{2}\theta \cos \frac{1}{2}\theta \\ 1= & {} \cos ^2\frac{1}{2}\theta + \sin ^2\frac{1}{2}\theta \\ \frac{d}{d\theta } \tan \theta= & {} \frac{1}{\cos ^2\theta } \end{aligned}$$

allow us to rewrite (6.70) as

6.72 $$\begin{aligned} g(x,E)= & {} g(x,E_0) \frac{\cos ^2\frac{1}{2}\beta \tau (x)}{\frac{1}{2}\beta }\Big (\frac{1}{2}\beta (u_2(E)+c_1(E)) \nonumber \\&+\beta c_2(E)\tan \frac{1}{2}\beta \tau (x) + \frac{1}{2}\beta (u_2(E)-c_1(E))\Big (\tan \frac{1}{2}\beta \tau (x)\Big )^2\Big ),\nonumber \\ \end{aligned}$$

which is of the form specified in $F_3$ with

6.73 $$\begin{aligned} \zeta (x) = \tan \frac{1}{2}\beta \tau (x) \end{aligned}$$

because

$$\begin{aligned} \zeta '(x)=\frac{\frac{1}{2}\beta \tau '(x)}{\cos ^2\frac{1}{2}\beta \tau (x)} = \frac{\frac{1}{2}\beta }{g(x,E_0)\cos ^2\frac{1}{2}\beta \tau (x)}. \end{aligned}$$

Using $\alpha =i\beta$ and (6.69) we rewrite (6.64) as

$$\begin{aligned} \widetilde{\mu }\left( \tau ^{-1}(t),E\right) = d_1(E)\cos \beta t + d_2(E)\sin \beta t + u_5(E)+\mathrm{Re}\, \lambda _1 \widetilde{g}(t,E) \end{aligned}$$

with

6.74 $$\begin{aligned} u_5(E):= & {} u_4(E)-\mathrm{Re}\, \lambda _1 u_2(E), \nonumber \\ d_1(E):= & {} \frac{k-1}{2}\beta c_2(E), \nonumber \\ d_2(E):= & {} -\frac{k-1}{2}\beta c_1(E). \end{aligned}$$

Equivalently we can write

$$\begin{aligned}&\widetilde{\mu }\left( \tau ^{-1}(t),E\right) \\&\quad =\cos ^2 \frac{1}{2}\beta t\left( u_5(E)+d_1(E)+2d_2(E)\tan \frac{1}{2}\beta t\right. \\&\qquad \left. +(u_5(E)-d_1(E))\left( \tan \frac{1}{2}\beta t\right) ^2\right) \\&\qquad +\mathrm{Re}\, \lambda _1 \widetilde{g}(t,E), \end{aligned}$$

which, in combination with (6.25) and (6.30) yields

6.75 $$\begin{aligned} {\mu }\left( x,E\right)= & {} \frac{\mu (x,E_0)+\mathrm{Re}\,\lambda _1}{g(x,E_0)}g(x,E) \nonumber \\&+\frac{1}{1+\zeta (x)^2}\left( u_5(E)+d_1(E)+2d_2(E)\zeta (x)\right. \nonumber \\&\left. +(u_5(E)-d_1(E))\zeta (x)^2 \right) , \end{aligned}$$

where $\zeta$ is defined in (6.73). Thus our task is find $\gamma _0(x)$ and $\mu _0(E)$ such that

6.76 $$\begin{aligned} \mu (x,E) =\gamma _0(x)g(x,E)+\mu _0(E)+(k-1)\frac{1}{2}\beta (u_2(E)-c_1(E))\zeta (x). \end{aligned}$$

The right hand sides of (6.75) and (6.76) are equal if and only

6.77 $$\begin{aligned}&\frac{1}{1+\zeta (x)^2}\left( u_5(E)+d_1(E)+2d_2(E)\zeta (x)+(u_5(E)-d_1(E))\zeta (x)^2 \right) \nonumber \\&\quad =\frac{\widetilde{\gamma }_0(x)}{1+\zeta (x)^2}\left( \frac{1}{2}\beta (u_2(E)+c_1(E))+\beta c_2(E)\zeta (x)\right. \nonumber \\&\qquad \left. +\frac{1}{2}\beta (u_2(E)-c_1(E))\zeta (x)^2\right) \nonumber \\&\qquad +\mu _0(E)+(k-1)\frac{1}{2}\beta (u_2(E)-c_1(E))\zeta (x), \end{aligned}$$

where we have replaced the unknown function $\gamma _0$ by a new unknown function $\widetilde{\gamma }_0$ via the relation

6.78 $$\begin{aligned} \gamma _0(x) =\frac{\mu (x,E_0)+\mathrm{Re}\, \lambda _1}{g(x,E_0)} + \frac{\frac{1}{2}\beta \widetilde{\gamma }_0(x)}{g(x,E_0)} \end{aligned}$$

and where we used (6.72), (6.73) and the identity $\left( 1+\zeta (x)^2\right) \cos ^2\frac{1}{2}\beta \tau (x) =1$.

We rewrite the left hand side of (6.77) as

$$\begin{aligned} u_5(E)+d_1(E)+\frac{2\zeta (x)}{1+\zeta (x)^2}\left( d_2(E)-d_1(E)\zeta (x)\right) \end{aligned}$$

and choose $\mu _0(E)=u_5(E)+d_1(E)$. This leaves us with

$$\begin{aligned}&\frac{2\zeta (x)}{1+\zeta (x)^2}(d_2(E)-d_1(E)\zeta (x)) \\&\quad =\frac{\widetilde{\gamma }_0(x)}{1+\zeta (x)^2}\left( \frac{1}{2}\beta (u_2(E)+c_1(E))+\beta c_2(E)\zeta (x)+ \frac{1}{2}\beta (u_2(E)-c_1(E))\zeta (x)^2\right) \\&\qquad +(k-1)\frac{1}{2}\beta (u_2(E)-c_1(E))\zeta (x). \end{aligned}$$

Hence we should have

$$\begin{aligned} \frac{1}{\zeta (x)}\widetilde{\gamma }_0(x)= & {} \frac{2d_2(E)-2d_1(E)\zeta (x)-(k-1)\frac{1}{2}\beta (u_2(E)-c_1(E))\left( 1+\zeta (x)^2\right) }{\frac{1}{2}\beta (u_2(E)+c_1(E))+\beta c_2(E)\zeta (x)+\frac{1}{2}\beta (u_2(E)-c_1(E))\zeta (x)^2} \\= & {} \frac{2d_2(E)-(k-1)\frac{1}{2}\beta (u_2(E)-c_1(E))-2d_1(E)\zeta (x)}{\frac{1}{2}\beta (u_2(E)+c_1(E))+\beta c_2(E)\zeta (x)+\frac{1}{2}\beta (u_2(E)-c_1(E))\zeta (x)^2} \\&+\frac{-(k-1)\frac{1}{2}\beta (u_2(E)-c_1(E))\zeta (x)^2}{\frac{1}{2}\beta (u_2(E)+c_1(E))+\beta c_2(E)\zeta (x)+\frac{1}{2}\beta (u_2(E)-c_1(E))\zeta (x)^2} \\= & {} -(k-1), \end{aligned}$$

where the last equality is a consequence of (6.74). We conclude that $\mu$ is of the form specified in $F_3$ with

6.79 $$\begin{aligned} \mu _0(E)= & {} u_5(E)+d_1(E) \nonumber \\ \gamma _0(x)= & {} \frac{\mu (x,E_0)+\mathrm{Re}\,\lambda _1-\frac{1}{2}\beta (k-1)\zeta (x)}{g(x,E_0)}. \end{aligned}$$

The next task is to specify a *real* matrix $H(E_0)$ with eigenvalues $\lambda _j$ given by (6.69). From (6.72) we deduce that necessarily $c_1(E_0)=c_2(E_0)=0$ and $u_2(E_0)=1$. This translates into $v_2(E_0)=0,\, v_1(E_0)=v_3(E_0)=\frac{1}{2}\beta$ for the parameters $v_i,\, i=1,2,3$ in $F_3$. So if we put $H(E_0) = H_0(E_0) - \mathrm{Re}\, \lambda _1I$, then the formulation of $F_3$ suggests to consider

6.80 $$\begin{aligned} H_0(E_0) = \frac{1}{2}\beta M, \end{aligned}$$

where *M* is the $k \times k$-matrix with entries

6.81 $$\begin{aligned} M_{\ell j} = \left\{ \begin{array}{ll} -(k-\ell ) &{}\quad \mathrm{if}\; j=\ell +1, \\ j &{}\quad \mathrm{if} \;\ell =j+1, \\ 0&{}\quad \mathrm{otherwise.} \end{array} \right. \end{aligned}$$

In the present context the key point is to find a solution to the linear ODE system corresponding to *M*. The result is given in the following lemma.

#### Lemma 6.13

Define the *k*-vector $\widetilde{\varPhi }(t)$ by

6.82 $$\begin{aligned} \widetilde{\varPhi }_j(t) = \sin ^{j-1} t \, \cos ^{k-j} t, \quad j=1,2,\ldots ,k. \end{aligned}$$

Then

6.83 $$\begin{aligned} \frac{d}{dt}\widetilde{\varPhi }(t) = M\widetilde{\varPhi }(t). \end{aligned}$$

#### Proof

The *j*th component of both sides equals

$$\begin{aligned} (j-1)\widetilde{\varPhi }_{j-1}(t) - (k-j)\widetilde{\varPhi }_{j+1}(t) \end{aligned}$$

(with the convention that zero times the undefined $\widetilde{\varPhi }_0$ or $\widetilde{\varPhi }_{k+1}$ equals zero). $\square$

In order to determine the eigenvalues of *M* we shall relate *M* to the Kac-Sylvester matrix *K* defined by

6.84 $$\begin{aligned} K_{\ell j}=\left\{ \begin{array}{ll} \ell &{}\quad \mathrm{if} \; j=\ell +1, \\ k-j &{}\quad \mathrm{if} \; \ell =j+1, \\ 0 &{}\quad \mathrm{otherwise.} \end{array} \right. \end{aligned}$$

It is known (Taussky and Todd 1991) that the eigenvalues of *K* are given by

6.85 $$\begin{aligned} \lambda _j^K = 2j-k-1, \quad j=1,2,\ldots ,k. \end{aligned}$$

In fact much more is known and we recommend the reader to have a look at the paper by Taussky and Todd (1991).

#### Lemma 6.14

Let *S* be the $k \times k$ diagonal matrix with complex diagonal entries

6.86 $$\begin{aligned} S_{jj} = i^{j-1}, \quad j=1,2,\ldots , k. \end{aligned}$$

Then

6.87 $$\begin{aligned} SMS^{-1} = iK^T. \end{aligned}$$

#### Proof

$S^{-1}$ is the diagonal matrix with entries

$$\begin{aligned} \left( S^{-1}\right) _{jj} = (-i)^{j-1} \end{aligned}$$

and hence

$$\begin{aligned} \left( SMS^{-1}\right) _{\ell j} =\sum _{m=1}^k S_{\ell m} \sum _{n=1}^k M_{mn} \left( S^{-1}\right) _{nj} =i^{\ell -1}M_{\ell j}(-i)^{j-1}. \end{aligned}$$

If $j=\ell +1$, then $i^{\ell -1} (-i)^{j-1} =i^{-1}=-i$ and accordingly

$$\begin{aligned} \left( SMS^{-1}\right) _{\ell j} = i(k-\ell ) = iK_{j\ell }. \end{aligned}$$

If $\ell =j+1$, then $i^{\ell -1} (-i)^{j-1} =(-i)^{-1}=i$ and in this case

$$\begin{aligned} \left( SMS^{-1}\right) _{\ell j} = ij = iK_{j\ell }. \end{aligned}$$

$$\square $$

#### Corollary 6.15

The eigenvalues of *M* are given by

6.88 $$\begin{aligned} \lambda _j^M = (2j-k-1)i, \quad j=1,2,\ldots ,k. \end{aligned}$$

Motivated by (6.69), (6.80) and Lemma 6.13 we now choose

6.89 $$\begin{aligned} H(E_0) = H_0(E_0) + \mathrm{Re}\,\lambda _1 I= \frac{1}{2}\beta M + \mathrm{Re}\,\lambda _1 I \end{aligned}$$

and

6.90 $$\begin{aligned} \varPhi (t) = \widetilde{\varPhi }\left( \frac{1}{2}\beta t\right) e^{\mathrm{Re}\, \lambda _1 t}. \end{aligned}$$

Combining (6.90) with (6.23) and (6.28) we obtain

6.91 $$\begin{aligned} w(x) = e^{\int _{x_b}^x \frac{\mu (y,E_0)}{g(y,E_0)}dy} \widetilde{\varPhi }\left( \frac{1}{2}\beta \tau (x) \right) e^{\mathrm{Re}\,\lambda _1 \tau (x)}. \end{aligned}$$

Since $v_2(E_0)=0$ and $v_1(E_0) = v_3(E_0)=\frac{1}{2}\beta$ we have

$$\begin{aligned} g(x,E_0)=v_0(x)\frac{1}{2}\beta \left( 1+\zeta (x)^2 \right) =\frac{1}{2}\beta \frac{1+\zeta (x)^2}{\zeta '(x)}. \end{aligned}$$

To see that

$$\begin{aligned} \mu (x,E_0) = \gamma _0(x)g(x,E_0) - \mathrm{Re}\, \lambda _1 +\frac{1}{2}\beta (k-1)\zeta (x) \end{aligned}$$

we first deduce from (6.79) that

$$\begin{aligned} \mu _0(E_0) = u_5(E_0) + d_1(E_0). \end{aligned}$$

next from (6.74) that $d_1(E_0)=0$ and

$$\begin{aligned} u_5(E_0)=u_4(E_0)-\mathrm{Re}\,\lambda _1 u_2(E_0) \end{aligned}$$

and from (6.64) and (6.26) that $u_4(E_0)=0$ and, as above, from (6.72) that $u_2(E_0)=1$.

So

$$\begin{aligned} \int _{x_b}^x \frac{\mu (y,E_0)}{g(y,E_0)}dy= & {} \int _{x_b}^x\gamma _0(y)dy +\int _{x_b}^x\frac{-\mathrm{Re}\,\lambda _1+\frac{1}{2}\beta (k-1)\zeta (y)}{\frac{1}{2}\beta \left( 1+\zeta (y) \right) ^2}\zeta '(y)dy \\= & {} \int _{x_b}^x\gamma _0(y)dy - \mathrm{Re}\,\lambda _1 \tau (x) + \frac{k-1}{2}\log \left( 1+\zeta (x)^2 \right) , \end{aligned}$$

where we have used the fact that, on account of (6.73),

$$\begin{aligned} \frac{\zeta '(y)}{1+\zeta (y)^2} = \frac{1}{2}\beta \tau '(y) \end{aligned}$$

and that

$$\begin{aligned} \frac{\zeta (y)\zeta '(y)}{1+\zeta (y)^2} = \frac{d}{dy} \frac{1}{2} \log \left( 1+\zeta (y)^2\right) . \end{aligned}$$

Hence (6.91) can be written in the form

$$\begin{aligned} w(x) = e^{\int _{x_b}^x\gamma _0(y)dy} \left( 1+\zeta (x)^2 \right) ^{\frac{k-1}{2}} \widetilde{\varPhi }\left( \frac{1}{2}\beta \tau (x)\right) \end{aligned}$$

and, using (6.73) once more, as

$$\begin{aligned} w(x) = e^{\int _{x_b}^x\gamma _0(y)dy}\frac{1}{\cos ^{k-1} \frac{1}{2}\beta \tau (x)} \widetilde{\varPhi }\left( \frac{1}{2}\beta \tau (x)\right) . \end{aligned}$$

Recalling (6.82) we conclude that

6.92 $$\begin{aligned} w_j(x) = e^{\int _{x_b}^x\gamma _0(y)dy}\left( \tan \frac{1}{2}\beta \tau (x) \right) ^{j-1} \end{aligned}$$

exactly as specified in $F_3$ when $\zeta$ is given by (6.73).

Finally we observe that once $g(x,E),\, \mu (x,E)$ and *w*(*x*) have been determined, we choose *H*(*E*) such that (4.33) is indeed an identity. This leads to the matrix *H*(*E*) as specified in $F_3$.

We have thus reached the end of the proof of Theorem 5.3.

## Applications to age- and stage-structured population models

In this section we illustrate our general results by giving a characterisation of ODE-reducible age-structured models and by giving conditions for when the ODE-reduction of a structured model could be interpreted biologically in terms of physiological stages.

Age is a peculiar i-state. It is characterised by two properties: age advances equally with time, so the growth rate *g* is identically equal to 1, and all individuals are born with the same i-state $x_b=0$. The latter property means that the reproduction rate has the form

7.1 $$\begin{aligned} \beta (x,E,\cdot ) = \widetilde{\beta }(x,E)\delta _0, \end{aligned}$$

that is, its range in the space of measures is the one-dimensional subspace spanned by $\delta _0$.

Incidentally, please note that these two properties guarantee that the p-state has a density $n(t,\cdot )$ at time $t >s$ if it has a density at time *s* and that the evolution in time of this density is governed by the PDE

7.2 $$\begin{aligned} \frac{\partial }{\partial t} n(t,x) + \frac{\partial }{\partial x} n(t,x) = -\mu (x,E(t))n(t,x) \end{aligned}$$

with boundary condition

7.3 $$\begin{aligned} n(t,0) = \int _0^\infty \widetilde{\beta }(x,E(t)) n(t,x) dx. \end{aligned}$$

So here we are, in the spirit of Gurtin and MacCamy (1974, 1979a, b), Murphy (1983) and many others, classifying when the problem (7.2) and (7.3) reduces to a system of ODEs, but with two additional features:

- we consider explicitly output, that is, we specify what properties of the solutions matter;
- we want to derive conditions that are not only sufficient, but in fact necessary if reproduction is part of the output.

As explained in Sect. 4, the structured population model is ODE-reducible if the transport-degradation model is ODE-reducible and the reproduction process is such that there is a $k \times k$ matrix *M*(*E*) such that (4.28) holds. The one-dimensional range property leads to simplifications, which we will now describe.

With (7.1) the restriction (4.28) becomes

7.4 $$\begin{aligned} \widetilde{\beta }(x,E)w(0) = M(E)w(x), \end{aligned}$$

or written out for each row $i=1,2,\ldots ,k$,

7.5 $$\begin{aligned} \widetilde{\beta }(x,E)w_i(0) = M_{i1}(E)w_1(x) + M_{i2}(E)w_2(x)+\cdots +M_{ik}(E)w_k(x). \end{aligned}$$

It follows that

7.6 $$\begin{aligned} \widetilde{\beta }(x,E)= \sum _{j=1}^k \widetilde{\beta }_j(E)w_j(x) \end{aligned}$$

with

7.7 $$\begin{aligned} \widetilde{\beta }_j(E)= \frac{M_{ij}(E)}{w_i(0)} \end{aligned}$$

independent of *i*, that is, the elements $M_{ij}(E)$ of the matrix *M*(*E*) are

7.8 $$\begin{aligned} M_{ij}(E)=w_i(0)\widetilde{\beta }_j(E) \end{aligned}$$

and thus *M*(*E*), too, has one-dimensional range (namely the subspace spanned by $(w_1(0),\, w_2(0), \ldots ,\, w_k(0))^T$).

Next we show that for age structured models the family $F_3$ is included in family $F_2$.

Because $g=1$ in age-structured models, the entries for *g* in the families $F_2$ and $F_3$ imply that the functions $v_1,\, v_2$ and $v_3$ are constants. In the case of family $F_2$ it follows that also $v_0$ is a constant, which we without loss of generality take equal to 1 and so

7.9 $$\begin{aligned} \zeta (x) =\int _0^x \frac{dy}{v_0(y)} =x. \end{aligned}$$

On the other hand, in the case of family $F_3$, the entry for *g* and the fact that $\zeta '(x)=1/v_0(x)$ and $g=1$ imply that $\zeta (x)$ is the unique solution of the initial value problem

7.10 $$\begin{aligned} \zeta '(x) = v_1 + v_2\zeta (x) + v_3\zeta (x)^2, \quad \zeta (0)=0. \end{aligned}$$

We now deduce from the entries for $\mu$ that in all three families the death rate $\mu (x,E)$ is a sum of two functions, one depending only on *x* and the other depending only on *E*:

7.11 $$\begin{aligned} \mu (x,E) = \gamma _0(x) + \mu _0(E), \end{aligned}$$

in the case of family $F_2$ and

7.12 $$\begin{aligned} \mu (x,E) = \left( \widetilde{\gamma }_0(x) + (k-1)v_3\zeta (x)\right) + \mu _0(E), \end{aligned}$$

in the case of family $F_3$. In (7.12) we have added a tilde to the $\gamma _0$ parameter in family $F_3$. The reason for this will become clear soon.

The next lemma gives the relationship between the families $F_2$ and $F_3$.

### Lemma 7.1

The $F_2$ member with $g=1,\, \zeta (x)=x, \varLambda = H_0$ and $\mu (x,E)$ given by (7.11) coincides with the $F_3$ member with $g=1$, $\zeta$ the solution of (7.10) and $\mu (x,E)$ given by (7.12) with

7.13 $$\begin{aligned} \widetilde{\gamma }_0(x)= \gamma _0(x) - (k-1)v_3\zeta (x). \end{aligned}$$

### Proof

Take *w* from the family $F_3$:

$$\begin{aligned} w_j(x) = e^{\int _0^x \widetilde{\gamma }_0(y) dy} \zeta (x)^{j-1}, \quad j=1,2,\ldots ,k. \end{aligned}$$

Then

7.14 $$\begin{aligned} w_j'(x)= & {} \widetilde{\gamma }_0(x)w_j(x)+(j-1)w_{j-1}(x)\zeta '(x) \nonumber \\= & {} \gamma _0(x)w_j(x) +(j-1)w_{j-1}(x)(v_1+v_2\zeta (x)) \nonumber \\&-(k-1)v_3\zeta (x)w_j(x)+(j-1)v_3w_{j-1}(x)\zeta (x)^2. \end{aligned}$$

Now note that

$$\begin{aligned} \zeta (x) w_j(x) = \zeta (x)^2w_{j-1}(x) \end{aligned}$$

and that this quantity equals $w_{j+1}(x)$ if $j \le k-1$. For $j=k$ the last two terms of (7.14) cancel. This shows that *w* satisfies the equation

$$\begin{aligned} w'(x) = \left( \gamma _0(x)I +H_0\right) w(x), \end{aligned}$$

that is, *w* is from family $F_2$ with $\varLambda = H_0$. $\square$

By Lemma 7.1 it suffices to concentrate on family $F_2$ of the catalogue (the scalar representation of family $F_1$ is a very special case of this). We have the following result:

### Theorem 7.2

Under the restriction that the birth rate is of the form (7.6), an age-structured population model is ODE-reducible if and only if the death rate can be written as (7.11) and the *w* occurring in (7.6) has the form

7.15 $$\begin{aligned} w(x) =e^{\int _0^x \gamma _0(y)dy} e^{x\varLambda } w(0) \end{aligned}$$

for some $k \times k$ matrix $\varLambda$ and *w*(0) satisfying (*H2*) and (*H3*).

When this is the case,

7.16 $$\begin{aligned} N(t) = \int _0^\infty w(x)m(t,dx) = \int _0^\infty w(x)n(t,x)dx \end{aligned}$$

satisfies the ODE

7.17 $$\begin{aligned} \frac{d}{dt}N(t) = \left( M(E(t)) + \varLambda -\mu _0(E(t))\right) N(t), \end{aligned}$$

with *M*(*E*) defined by (7.8).

Once *N* has been solved from (7.17), the population density is recovered from the explicit formula

7.18 $$\begin{aligned} n(t,x) = e^{-\int _0^x \gamma _0(y)dy} e^{-\int _{t-x}^t \mu _0(E(\tau ))d\tau } \sum _{j=1}^k \widetilde{\beta }_j(E(t-x))N_j(t-x), \quad x<t.\nonumber \\ \end{aligned}$$

Age-structured models can also be written directly as scalar renewal equations (with input dependent kernel) for the population birth rate. In an earlier paper (Diekmann et al. 2018) we derived necessary and sufficient conditions for ODE-reducibility of such renewal equations. When applied to age-structured models the results of the two papers are of course the same.

Let us point out that the ODE (7.17) resulting from the reduction does not necessarily have any biological interpretation. But if $\varLambda$ is a transition matrix, then the indices of *N* could be interpreted as discrete states. In particular, if $\varLambda$ has negative entries on the diagonal and corresponding positive entries on the subdiagonal, then the states form a linear chain and the states can be interpreted as stages (Nisbet and Gurney 1983; Nisbet et al. 1985, 1986; Thieme 2003) if, in addition, $w_j(0)=0$ for $j\ge 2$, which means that all newborns have stage $j=1$. This explains why MacDonald (1978, 1989) called ODE-reduction the *linear chain trick*. The classical example of such linear chain trickery is obtained by choosing $\gamma _0=0$, and $w_j$ the Erlang distribution (a special case of the gamma distribution)

7.19 $$\begin{aligned} w_j(x) = \frac{\alpha ^j}{(j-1)!} x^{j-1}e^{-\alpha x}, \end{aligned}$$

and $\varLambda$ a matrix with diagonal elements $-\alpha$ and subdiagonal elements $+\alpha$. The ODE (7.17) then becomes

7.20 $$\begin{aligned} \frac{d}{dt}N_1(t)= & {} \alpha \sum _{j=1}^k \widetilde{\beta }_j(E) N_j -(\alpha + \mu _0(E))N_1, \end{aligned}$$

7.21 $$\begin{aligned} \frac{d}{dt}N_i(t)= & {} \alpha N_{i-1} -(\alpha + \mu _0(E))N_i, \quad i=2,3,\ldots ,k, \end{aligned}$$

clearly showing the stage structure (Gyllenberg 2007).

## Discussion

Our focus should be on the biological problem: when is a physiologically structured model ODE representable? That is the question that matters, both practically and foundationally, for community modelling. In the wake of an answer we can re-examine the community models that are directly formulated in terms of ODE in order to determine what i-level physiological processes are, or are not, incorporated.

If we are prepared to specify beforehand a set of $k_0$ linearly independent output functionals $P_i^0$ that should be computable from the ODE variables, we can perform a test. The test was explained in Sects. 2.1.4 and 4.1, but we repeat it here while employing, exactly as in (2.18), the short hand of an abstract ODE

8.1 $$\begin{aligned} \frac{d}{dt}n(t) = A(E(t)) n(t) \end{aligned}$$

rather than the constructively defined evolutionary system $U_E^c(t,s)$ of Sect. 3. Note that now we do *not* use the decomposition $A(E) = A_0(E) + B(E)$.

TEST: Let $$w_i^0$$ be the element of the dual space representing $$P_i^0$$. A first condition for ODE representability is that $$w_i^0$$ belongs to the domain of $$A(E)^*$$ for all *E* in $${\mathscr {E}}$$. If $$A(E)^*w_i^0$$ belongs to the subspace spanned by $$\{w_1^0, w_2^0\ldots ,w_{k_0}^0\}$$ for all *i* and all *E*, the family *A*(*E*) is ODE-reducible. If the subspace spanned by $$\{w_1^0, w_2^0\ldots ,w_{k_0}^0\}$$ and the $$A(E)^*w_i^0$$ for all *i* and all *E* is infinite dimensional, the family *A*(*E*) is not ODE-reducible. In case this subspace has dimension $$k_1$$, with $$k_1$$ finite but larger than $$ k_0$$, choose a basis consisting of $$w_i^1$$ with $$i=1,\ldots ,k_1$$ and $$w_i^1=w_i^0$$ for $$i=1,2,\ldots ,k_0$$ and repeat this procedure with the superscript 0 replaced by the 1. The procedure can be repeated. If the procedure terminates after a finite number of steps with a finite dimensional subspace, the family *A*(*E*) is ODE-reducible.

Whenever biological knowledge and interest suggest a meaningful collection of $w_i^0$, one should perform this test. Clearly this requires context specific information and does not lead to an explicit catalogue. In this paper we concentrated on a special subclass of physiologically structured models for which we could obtain detailed results. The description of the subclass is based on the decomposition $A(E) = A_0(E) + B(E)$, with $A_0(E)$ capturing transport and degradation and *B*(*E*) capturing reproduction. Our main result provides a complete catalogue of ODE reducible families $A_0(E)$. The catalogue lists conditions on the growth rate *g* and the mortality rate $\mu$ and provides explicit expressions for the functionals that define the components of the ODE. Once these functionals are known, one can test whether or not the subspace that they span is left invariant by $B(E)^*$ for all *E* in ${\mathscr {E}}$. If the subspace is invariant, the family *A*(*E*) is ODE-reducible. Moreover, the asymptotic population dynamics is in that case fully determined by the ODE (recall (2.27) and Proposition 7.2).

So the catalogue is complete for a restricted class of structured population models. We now provide some examples of biologically relevant ODE-reducible models that do *not* belong to that particular restricted class, because the reproduction process does not satisfy condition (4.28). Our first example shows how the simple model of Example 1.1 can be treated.

### Example 8.1

Assume a one-dimensional i-state space and that $\beta$ is of the form (4.32). Equation (4.27) then becomes

8.2 $$\begin{aligned} g(x,E) w'(x) -\mu (x,E)w(x) + \widetilde{\beta }(x,E)\int _\varOmega w(y)\beta _0(dy) = K(E)w(x). \end{aligned}$$

For definiteness, we normalise $\beta _0$ by

8.3 $$\begin{aligned} \int _\varOmega \beta _0(dy) = 1. \end{aligned}$$

If we are seeking a scalar representation (*w* is a scalar valued function), then the condition (8.2) transforms into

8.4 $$\begin{aligned} \frac{g(x,E) w'(x) + \widetilde{\beta }(x,E)\int _\varOmega w(y)\beta _0(dy)}{w(x)} -\mu (x,E) = K(E). \end{aligned}$$

If the output is the total population, then $w(x)=1$ and (8.4) becomes

8.5 $$\begin{aligned} \widetilde{\beta }(x,E) -\mu (x,E) = K(E). \end{aligned}$$

If the output is total biomass, then $w(x)=x$ and (8.4) becomes

8.6 $$\begin{aligned} \frac{g(x,E) + \widetilde{\beta }(x,E)\int _\varOmega y\beta _0(dy)}{x} -\mu (x,E) = K(E). \end{aligned}$$

Note that the scalar factor $\int _\varOmega y\beta _0(dy)$ is the average weight of a newborn. In the case of one fixed size $x_b$ at birth, $\beta _0 = \delta _{x_b}$ and (8.6) becomes the same as condition (1.1).

We now provide some other examples that are ODE-reducible, but do not satisfy the restrictions of the present paper.

### Example 8.2

Let $\varOmega$ be a subset of the positive cone ${\mathbb {R}}^2_+$ and let

8.7 $$\begin{aligned} g(x,E) = \left( \begin{array}{c} a(E) +b(E)x_1 \\ c(E) \end{array}\right) . \end{aligned}$$

Then

8.8 $$\begin{aligned} (Dw)(x)g(x,E) = L(E)w(x) \end{aligned}$$

for the choice

8.9 $$\begin{aligned} L(E) = \left( \begin{array}{cccccc} 0 &{}0 &{}0&{}0&{}0&{}0\\ a(E) &{}b(E) &{}0&{}0&{}0&{}0 \\ 0 &{}2a(E) &{}2b(E) &{}0&{}0&{}0\\ 0&{}0&{}0 &{}-\kappa c(E) &{}0&{}0 \\ 0&{}0&{}0 &{}a(E) &{}b(E)-\kappa c(E) &{}0 \\ 0&{}0&{}0&{}0 &{}2a(E) &{}2b(E)-\kappa c(E) \end{array}\right) \end{aligned}$$

and

8.10 $$\begin{aligned} w(x) = \left( \begin{array}{c} 1 \\ x_1 \\ x_1^2\\ e^{-\kappa x_2} \\ x_1 e^{-\kappa x_2} \\ x_1^2 e^{-\kappa x_2} \end{array}\right) . \end{aligned}$$

So if

8.11 $$\begin{aligned} \mu (x,E) = \mu _0(E) \end{aligned}$$

and

8.12 $$\begin{aligned} \beta (x,E,\omega ) = f(E)\left( 1-e^{-\kappa x_2}\right) x_1^2 m_b(\omega ) \end{aligned}$$

with $m_b$ a probability measure on $\varOmega$, then

8.13 $$\begin{aligned}&(Dw(x))g(x,E) -\mu (x,E)w(x) +\int _\varOmega w(y) \beta (x,E,dy) \nonumber \\&\quad =L(E)w(x) -\mu _0(E)w(x) +f(E)(w_3(x)-w_6(x))\int _\varOmega w(y)m_b(dy)\nonumber \\ \end{aligned}$$

and hence (4.27) is satisfied with

8.14 $$\begin{aligned} K(E) = L(E)-\mu _0(E)I +f(E)M, \end{aligned}$$

where *M* is the $6\times 6$ matrix with entries

8.15 $$\begin{aligned} M_{ij} := \int _\varOmega w_i(y) m_b(dy) \left( \delta _{3j}-\delta _{6j} \right) . \end{aligned}$$

Here $\delta$ is the Kronecker $\delta$, that is, $\delta _{ij}=1$ if $i=j$ and zero otherwise. In words: *M* is the matrix with only two non-zero columns, the third and the sixth, and on the *i*th row the element equals $\int _\varOmega w_i(y)m_b(dy)$ in column 3 and $-\int _\varOmega w_i(y)m_b(dy)$ in column 6.

The biological interest of Example 8.2 is that if we let the measure $m_b$ be concentrated on the axis $x_2=0$, we may interpret $x_1$ as size and $x_2$ as physiological age. The rate of giving birth is proportional to the square of the individual’s size with the proportionality coefficient a function of physiological age that increases monotonically from zero at zero to a finite limit depending on the environmental condition.

The next two examples are not biologically motivated, but are intended to illustrate that, even after scaling of the i-state variable, the growth rate is not restricted to a linear dependence on the i-state components (recall that in our catalogue only $F_3$ allows for a quadratic dependence on the i-state variable, but only so if the death rate $\mu$ contains a *k*-specific compensating term).

### Example 8.3

Let $\varOmega \subset {\mathbb {R}}^2$ and let

8.16 $$\begin{aligned} g(x,E) = \left( \begin{array}{c} a(E) +b(E)x_1 \\ c(E)x_1^2 \end{array}\right) . \end{aligned}$$

Then (8.8) holds for the choice

8.17 $$\begin{aligned} L(E) = \left( \begin{array}{cccc} 0 &{}0 &{}0&{}0\\ a(E) &{}b(E) &{}0&{}0 \\ 0 &{}2a(E) &{}2b(E) &{}0\\ 0&{}0 &{} c(E) &{}0 \end{array}\right) \end{aligned}$$

and

8.18 $$\begin{aligned} w(x) = \left( \begin{array}{c} 1 \\ x_1 \\ x_1^2 \\ x_2 \\ \end{array}\right) . \end{aligned}$$

### Example 8.4

Let $\varOmega \subset {\mathbb {R}}^3$ and let

8.19 $$\begin{aligned} g(x,E) = \left( \begin{array}{c} a_1(E) \\ a_2(E) \\ c_1(E) e^{\kappa _1x_1} +c_2(E) e^{\kappa _2x_2}+c_3(E) e^{\kappa _1x_1+\kappa _2x_2} \end{array}\right) . \end{aligned}$$

Then (8.8) holds for the choice

8.20 $$\begin{aligned} L(E) = \left( \begin{array}{cccc} \kappa _1 a_1(E) &{}0 &{}0&{}0\\ 0 &{}\kappa _2a_2(E) &{}0 &{}0\\ 0&{}0 &{} \kappa _1a_1(E)+\kappa _2a_2(E) &{}0 \\ c_1(E) &{}c_2(E) &{}c_3(E) &{}0 \end{array}\right) \end{aligned}$$

and

8.21 $$\begin{aligned} w(x) = \left( \begin{array}{c} e^{\kappa _1x_1} \\ e^{\kappa _2x_2} \\ e^{\kappa _1x_1+\kappa _2x_2}\\ x_3 \\ \end{array}\right) . \end{aligned}$$

### Example 8.5

Consider cells that split into two equal parts upon reaching a size $x_{\max }$. Assume that $g(x,E) = f(E)x$ and that $\mu (x,E)=\mu _0(E)$. Then the total mass *B* satisfies the ODE

$$\begin{aligned} \frac{dB}{dt}= f(E)B-\mu _0(E)B. \end{aligned}$$

If we start with a cohort (a Dirac mass in a point somewhere in the interval $(\frac{1}{2} x_{\max }, x_{\max })$), the distribution remains a cohort for all time, but the cohort jumps from $x_{\max }$ to $\frac{1}{2} x_{\max }$ every now and then, with the number of individuals in the cohort doubling whenever such a jump occurs (Huyer 1997). Note that when we change the reproduction rule and allow the two daughters to have slightly different sizes, the number of cohorts increases.

In an earlier paper (Diekmann et al. 2018) we gave a characterisation of ODE-reducibility for renewal equations of the form

8.22 $$\begin{aligned} b(t) = \int _{-\infty }^t K_E(t,s)b(s)ds, \end{aligned}$$

where the unknown *b* is an ${\mathbb {R}}^p$ valued function and the input-dependent kernel $K_E$ is a $p \times p$ matrix valued function. The result is that the renewal Eq. (8.22) with input is ODE-reducible if and only

8.23 $$\begin{aligned} K_E(t,s) =U(E(t))^T \varPhi _E(t,s)V(E(s)), \end{aligned}$$

where $\varPhi _E(t,s)$ is the fundamental matrix solution for the linear non-autonomous system of ordinary differential equations

8.24 $$\begin{aligned} \frac{d}{d\tau } Z(\tau ) = H(E(\tau )) Z(\tau ) \end{aligned}$$

for some matrix valued functions *H*, *U* and *V*. It should, however, be noted that in the earlier paper the requirement that the solution *b*(*t*) of the renewal Eq. (8.22) could be recovered from the solution *Z*(*t*) of the finite dimensional system (8.24) was built into the notion of ODE-reducibility, whereas we in the current paper only require that the *outputs* can be recovered (recall Fig. 2). More recently we have taken the abstract variant of (8.22) as the starting point for a discussion of ODE-reducibility. In the paper (Diekmann et al. 2019) we provide new examples, recap the present paper and explain the usefulness of *asymptotic* ODE-reducibility.
